# Integrating and visualizing primary data from prospective and legacy taxonomic literature

**DOI:** 10.3897/BDJ.3.e5063

**Published:** 2015-05-12

**Authors:** Jeremy A. Miller, Donat Agosti, Lyubomir Penev, Guido Sautter, Teodor Georgiev, Terry Catapano, David Patterson, David King, Serrano Pereira, Rutger Aldo Vos, Soraya Sierra

**Affiliations:** ‡ Naturalis Biodiversity Center, Leiden, Netherlands; § www.Plazi.org, Bern, Switzerland; | Pensoft, Sofia, Bulgaria; ¶ KIT / Plazi, Karlsruhe, Germany; # Pensoft Publishers, Sofia, Bulgaria; ¤ University of Sydney, Sydney, Australia; « The Open University, Milton Keynes, United Kingdom

**Keywords:** Araneae, Biodiversity informatics, Data mining, Open access, Spiders, Taxonomy, XML markup

## Abstract

Specimen data in taxonomic literature are among the highest quality primary biodiversity data. Innovative cybertaxonomic journals are using workflows that maintain data structure and disseminate electronic content to aggregators and other users; such structure is lost in traditional taxonomic publishing. Legacy taxonomic literature is a vast repository of knowledge about biodiversity. Currently, access to that resource is cumbersome, especially for non-specialist data consumers. Markup is a mechanism that makes this content more accessible, and is especially suited to machine analysis. Fine-grained XML (Extensible Markup Language) markup was applied to all (37) open-access articles published in the journal Zootaxa containing treatments on spiders (Order: Araneae). The markup approach was optimized to extract primary specimen data from legacy publications. These data were combined with data from articles containing treatments on spiders published in Biodiversity Data Journal where XML structure is part of the routine publication process. A series of charts was developed to visualize the content of specimen data in XML-tagged taxonomic treatments, either singly or in aggregate. The data can be filtered by several fields (including journal, taxon, institutional collection, collecting country, collector, author, article and treatment) to query particular aspects of the data. We demonstrate here that XML markup using GoldenGATE can address the challenge presented by unstructured legacy data, can extract structured primary biodiversity data which can be aggregated with and jointly queried with data from other Darwin Core-compatible sources, and show how visualization of these data can communicate key information contained in biodiversity literature. We complement recent studies on aspects of biodiversity knowledge using XML structured data to explore 1) the time lag between species discovry and description, and 2) the prevelence of rarity in species descriptions.

## Introduction

The limited accessibility of taxonomic literature is an impediment not only to taxonomic research but to the effective functioning of the biodiversity classification system that underlies biology (Godfray et al. 2007). Taxonomic literature contains not only the descriptions of biodiversity as we know it, but citations of the specimens that form the basis for this primary taxonomic study. Because of their contribution to revisions, monographs, descriptions, and other primary taxonomic literature, these specimen records are among the highest quality biodiversity data available (Meier and Dikow 2004, Dikow et al. 2009). In an age when we have the tools to effectively manage and analyze large quantities of data, and also when environmental changes call for data-driven decision-making, digitization, structuring and extraction of content from taxonomic literature is needed to provide a more comprehensive supply of information and an historical perspective to current societal issues involving the biosphere (Miller et al. 2012, Penev et al. 2011, Penev et al. 2010). For these data to be effective, they must be in a structured digital form that can be aggregated and universally queried. The value and relevance of aggregated primary biodiversity data is recognized by policy makers. Pro-iBiosphere (2012-2014, http://www.pro-ibiosphere.eu/) was an FP-7 (European Union Seventh Framework Programme, 2007-2013) funded coordination and policy development project to lay the groundwork for a unified digital biodiversity knowledge management system, including knowledge located in legacy literature. The European Biodiversity Observation Network (EU BON, 2012-2017, http://www.eubon.eu/​) is building a network to improve interoperability among biodiversity data to improve monitoring and assessment.​

To facilitate the aggregation and re-use of knowledge from taxonomic literature, the Swiss NGO Plazi (http://www.plazi.org/) has established a treatment bank that stores structured versions of taxonomic treatments marked up with XML (TaxonX schema, http://taxonx.org/). Taxonomic treatments are any descriptive or diagnostic contributions that add to our understanding of the taxonomy or nomenclature of that taxon (Penev et al. 2012, Penev et al. 2011, Agosti and Egloff 2009). Taxonomic publications typically contain one or more sections describing species or other taxa. These descriptions include a taxonomic name, references to previous descriptions of that taxon (whether referred to by that name or another) or an indication that the taxon is newly described, descriptive and diagnostic text sections, and notes on specimens, biology, ecology, distribution, and so on. Species-level treatments (and sometimes higher level treatments as well) are typically based on explicitly-cited specimens. Treatments are available directly from Plazi (http://plazi.cs.umb.edu/GgServer/search​), and are also shared externally with an assortment of cybertaxonomic resources including the Encyclopedia of Life (http://eol.org/), the Global Biodiversity Information Facility (http://www.gbif.org/), and Species-ID (http://species-id.net/wiki/). These sites harvest and display content according to their own model. This is accomplished using a variety of biodiversity informatics community standards including Darwin Core Archives (GBIF 2010). The XML-structuring of the content of the treatments allows them to be aggregated and reused according to diverse requirements. Pensoft (http://www.pensoft.net/about.php​) journals, which routinely use XML (TaxPub schema, an extension of the NLM DTD [National Library of Medicine, Document Type Definition], http://sourceforge.net/projects/taxpub/) to delimit semantic content, contribute taxonomic treatments to Plazi as part of their normal publishing workflow (Penev et al. 2012​). Treatments from legacy literature can be marked up and added to Plazi using the GoldenGATE XML markup editor (http://plazi.org/wiki/GoldenGATE_Editor​).

Fine-grained XML markup of legacy taxonomic literature allows us to experiment with new approaches to synthesizing the primary data that are the foundation of taxonomic research. During pro-iBiosphere’s Data Enrichment Hackathon (http://wiki.pro-ibiosphere.eu/wiki/Data_enrichment_hackathon,_March_17-21_2014; Vos et al. 2014), the capabilities of Plazi’s data search and retrieval system (SRS) were expanded. As a result, we can now produce a series of charts to represent and summarize the specimen data associated with any treatment or group of treatments. These charts contribute to dashboards that reveal, at a glance, key information of use to taxonomic researchers, collections managers, and other stakeholders. These include profiles of when specimens were collected (both by month of the year and by decade), specimens by elevation of collection site, proportions of male and female specimens, and specimens portioned by institutional collection, collector, and country of origin.

Spiders (Order: Araneae) are the seventh most speciose order of life on earth and an important megadiverse taxon in biodiversity studies (Miller et al. 2014a, Coddington et al. 2009​). Despite this, their online digital data presence is disproportionately small. Spiders are the largest taxon with a regularly updated online catalog; all treatments and their source publications are indexed in a single exhaustive resource - the World Spider Catalog (2015). This tool has promoted rigorous scholarship and amplified productivity in spider taxonomy. Until recently, the catalog was not structured in a way that allowed online databases and data aggregators to keep current. With the launch of the newly structured World Spider Catalog based in Bern, Switzerland, the time is ripe to move forward in how we present spider data on-line.

GBIF (the Global Biodiversity Information Facility - www.gbif.org) aggregates and serves occurrence data through a common portal. These data are applicable to a wide range of fields at the intersection of biodiversity, geography, and climate. To serve researchers in these fields requires that we plot species distributions, predict impacts from climate change, track invasive alien species, set informed conservation priorities, and more (http://www.gbif.org/usingdata/sciencerelevance). A leading mechanism for getting content into GBIF involves aggregating data from a network of large institutional collections, especially natural history collections. With more than half a billion records already available through GBIF, it might seem that a fine-grained markup process capable of adding occurrences is redundant. Yet, if we break down GBIF data by taxon, some strong patterns and biases emerge (Fig. 1). Birds (Class: Aves), which represent about 1% of animal species, are the subject of more than half of all GBIF records. However, most of these are observation records, not specimen-based records. It is the specimen-based records that are typically associated with vouchers in museum collections. Hymenoptera, the leader among diverse taxa in digital online specimen data, have about as many specimen records in GBIF as do birds, yet the diversity of Hymenoptera is an order of magnitude higher than birds (Hymenoptera: 122,767 species, http://hol.osu.edu/; Aves: 10,306 species, http://www.catalogueoflife.org/). Considering the number of species concerned, the amount of data for the world's most diverse taxa, including spiders, is comparatively low. This is not because megadiverse taxa are not well represented in the world’s natural history collections – far from it – it is because most of the specimen data are not available in structured digital form. Our capacity to call on data in digital form to address important questions (like setting wise conservation priorities or anticipating the effects of climate change) is limited by biases in the data that are currently available. The data currently available in GBIF clearly represent some taxonomic groups reasonably well. The complementary approach of using taxonomic literature as a source of specimen data may be more successful for megadiverse taxa.

In its short 14-year history, Zootaxa has published more articles on spider taxonomy than any other journal (World Spider Catalog 2014; Fig. 2a) and is a leading publisher of zoological taxonomy in general. It is an obvious place to explore how to structure legacy content for cybertaxonomic data aggregators. Open access publishing is broadly recognized as a means to ensure the wide dissemination and use of biodiversity knowledge for various scientific and policy applications (http://bouchoutdeclaration.org/; Thessen and Patterson 2011, Patterson et al. 2014, Hardisty et al. 2013). Zootaxa has a conditional open access policy where authors may choose to pay a fee to make their publications openly accessible under a Creative Commons attribution license (http://www.mapress.com/zootaxa/support/author.html, http://creativecommons.org/). Zootaxa began publishing articles with DOIs (digital object identifiers) in early 2013; Zootaxa has recently started assigning DOIs retroactively to all earlier articles (Table 1).

Pensoft was the first publisher to distribute semantic content to online taxonomic resources and databases as a routine part of the publication process, starting with Encyclopedia of Life (http://eol.org/) and ZooBank (http://www.zoobank.org/), then progressively expanding its content-sharing partners, now including Plazi (Penev et al. 2010​​). The newest offering from Pensoft, Biodiversity Data Journal, has implemented a more fine-grained semantic data model, including occurrence records parsed using Darwin Core (http://rs.tdwg.org/dwc/) fields (Smith et al. 2013). Like all Pensoft journals, Biodiversity Data Journal (BDJ) is open access, with all content freely available online. More than 90% of the treatments on spiders currently in Plazi come from three sources: Biodiversity Data Journal, followed by ZooKeys, then Zootaxa (mostly markup performed for this project). Occurence records are also mostly from Biodiversity Data Journal, with most of the remaining records from Zootaxa articles; records are not routinely parsed into fields for ZooKeys articles. We demonstrate here that semantically marked content from a prospective publication process can be seamlessly combined and analyzed along with data marked using GoldenGATE. ​

## Material and methods

We searched the online archive of Zootaxa publications for open access articles that included taxonomic treatments on spiders (Order: Araneae; http://www.mapress.com/zootaxa/taxa/Araneae.html). By the time the pro-iBiosphere project ended in August 2014, Zootaxa had published 531 articles on spiders, 45 (8.5%) of which were openly accessible. Eight of the open access articles contained no taxonomic treatments (e.g., were errata, editorials, obituaries, phylogenetic studies, etc.). The remaining 37 (7.0%) articles were marked up using the GoldenGATE Document Editor (Sautter 2013). PDF files were either read directly by GoldenGATE or first pre-processed using ABBYY FineReader 11. Articles were marked up using a series of pipelines customized for the pro-iBiosphere project.

Our markup approach structures data that relate to publications, treatments, and specimens. Data on publications include basic bibliographic information and the treatments they contain. Treatments are categorized according to taxonomic rank (species, genus, family, etc.), taxonomic status (new species, new combination, new genus, etc.), and taxonomic hierarchy. Sexually mature specimens are classified by gender (with other attributes available as relevant to taxonomic group, such as the caste for ants), collecting country, other locality-based fields, institutional collection code, type status (holotype, paratype, etc.), collector name, collection date, and elevation.

The Plazi srsStatCharts utility (http://plazi.cs.umb.edu/GgServer/srsStatCharts) was used to create a series of interactive dashboard pages to summarize these data. This utility works with the Google Visualization API (https://developers.google.com/chart/​). Source code for aggregation of XML structured treatment data on the Plazi server is available at https://code.google.com/p/goldengate-server-docs/source/browse/#git%2Fsrc%2Fde%2Fuka%2Fipd%2Fidaho%2FgoldenGateServer%2Fdcs​. The dashboard pages display a series of charts for various categories of data contained within the set of open access articles in Zootaxa and articles in Biodiversity Data Journal containing treatments on spiders. The dashboard pages were: 1) all treatments (Zootaxa), 2) species-rank treatments (Zootaxa), 3) all treatments (BDJ), 4) species-rank treatments (BDJ), 5) all treatments (Zootaxa + BDJ), 6) species-rank treatments (Zootaxa + BDJ) 7) one selected collection (California Academy of Sciences, CAS), 8) one selected collecting country (Russia), 9) one selected collector (Y. M. Marusik), 10) one selected article (Kronestedt and Marusik 2011), 11) one selected treatment (*Pardosa zyuzini* in Kronestedt and Marusik 2011), 12) one selected species (*Tenuiphantes tenuis*), 13) one selected author (Jeremy A. Miller). Dashboard pages are presented here as static graphics, but we recommend downloading the interactive pages (Suppl. materials 4, 5, 6, 7, 8, 9, 10, 11, 12, 13, 14, 15, 16) and viewing them in a browser (see Suppl. material 17​ for further information on how to construct and customize the javascript queries). Additional data analysis was performed in Microsoft Excel on data downloaded from Plazi's srsStats site (http://plazi.cs.umb.edu/GgServer/srsStats).

## Results

The dashboards are based on the 37 open access articles in Zootaxa containing treatments on spiders (Table 1), and five articles in Biodiversity Data Journal (Miller et al. 2013, Miller et al. 2014, Deltshev et al. 2013, Crespo et al. 2014, Čandek et al. 2013; note that data in Čandek et al. 2013 have been updated to reflect corrections made by the authors in a corrigendum, Čandek et al. 2015, Miller et al. 2015). The partitions of data offer novel ways to explore and visualize taxonomic research information from geographic, institutional, temporal, individual, and specimen-oriented perspectives. Below, we summarize data contained in the source articles. It is possible to create dashboards based on any institution, country, collector, author, article, treatment, taxonomic rank, type status, or combination of these, among other variables. ​

### Open access articles in Zootaxa containing treatments on spiders

The 37 open access Zootaxa articles on spiders contain 254 treatments, of which 212 are species treatments, based on 4,779 specimens (species treatments contain citations of 4,773 specimens, the difference in numbers is due to 26 specimens identified to genus only) (Fig. 3​). Articles contain a mean of 6.9 treatments (5.7 species-rank treatments) per publication and 22.5 specimens per species-rank treatment. 91 (42.9%) treatments are descriptions of new species. There are also 22 new combinations and three new genera. About a third of treatments (72 of all species treatments, 34 of new species descriptions) were based on a single specimen; the treatment with the most specimens had a total count of 851 (*Pardosa logunovi* in Kronestedt and Marusik 2011). The largest number of species treatments were from the family Agelenidae (28.3%, 60) but the largest number of specimens were Lycosidae (44.3%, 2,116). Adult males made up just over half (51.5%, 2,460) of the specimens, with the remainder divided between adult females (40.7%, 1943) and non-adults (7.8%, 370). The largest number of specimens was collected in Russia (34.8%, 1,660), which when combined with specimens from China (14.9%, 713) accounts for nearly half of the specimens cited. China was the country with specimens appearing in the most treatments (63); only six treatments included specimens from Russia. More than 17% of the specimens were not explicitly associated with an institutional collection (see below); the California Academy of Sciences was cited as the institution archiving the largest number of specimens (14.1%, 674), but Hunan Normal University is the repository for the largest number of primary type (holotype, syntype, lectotype, or neotype) specimens (23.4%, 49) and also had specimens featured in the largest number of treatments (48). Yuri Marusik was by far the most productive specimen collector, alone collecting 869 (19.5%) specimens, not counting the specimens he collected collaboratively with others. Tang Guo collecting alone contributed specimens to the most treatments (17), but Charles Griswold, working alone or with several collaborators, contributed specimens to more treatments (see Discussion: Tracking Individuals). The 1,007 specimens that had the elevation specified were collected across a wide range, with a few (six) collected between 4,001-4,500 m. Overall, most specimens of both sexes but especially males were collected in July, followed by June. The bulk of the specimens cited in this group of publications, which spanned 2002-2014, were collected in the 1990s and 2000s. 2010 was a year of peak activity both in terms of articles and treatments. Peter Jäger was the lead author on the most articles (13.5%, five), but Xin-Ping Wang was lead author on the most treatments (28.3%, 60) and Torbjörn Kronestedt was lead author documenting the most specimens (44.3%, 2,116).

### Biodiversity Data Journal articles containing treatments on spiders

The five spider articles published in Biodiversity Data Journal before August 2014 contain 742 treatments, of which 672 are species treatments, based on 3,432 specimens (species treatments contain citations of 3,399 specimens) (Fig. 4​). Four treatments in this set of articles concern insects (Miller et al. 2013​), the rest concern spiders; if desired, it would be simple to add a filter to show only spider treatments (&FILTER_tax.orderEpithet=Araneae; see Suppl. material 17​). Articles contain a mean of 148.4 treatments (134.4 species-rank treatments) per publication and 5.1 specimens per species-rank treatment. Most of these treatments are faunistic occurence records without descriptive information; one (0.1%) species-rank treatment is a description of a new species. There are no new combinations or descriptions of any new higher rank taxa. About 10% of all treatments (but only 1.3% of species treatments) are missing some higher taxonomic information; this is typically supplied not by the authors but by an automated database query that sometimes fails to interpret the author's intentions (see Döring 2015). 496 (73.8%) of the species treatments explicitly cited specimens. Of these, about two fifths (42.1%, 209) were based on a single specimen; the one new species was based on 69 specimens; the species with the most specimens (10.4%, 352) was *Tenuiphantes tenuis*, which appeared in three treatments (Čandek et al. 2013, Crespo et al. 2014, Deltshev et al. 2013). The dominant family by both treatments (22.9%, 154) and specimens (33.4%, 1,134) was the Linyphiidae. Adult females made up the majority (60.5%, 2,056) of specimens, with the remainder divided between adult males (38.9%, 1,323) and non-adults (0.6%, 20). The largest number of specimens was collected in Portuguese territory (Madeira island; 43.7%, 1,487). Slovenia was the country with specimens appearing in the most treatments (225); only 48 treatments included specimens from Portugal. Very few specimens (2.4%, 80) were explicitly associated with an institutional collection; these were divided among three collections (two institutions and one personal collection). The single holotype specimen was deposited at Universiti Malaysia Sabah's Institute for Tropical Biology and Conservation, Borneensis (UMS). The team of Kuntner, Gregoric, and Candek collected the largest number of specimen records (43.1%, 823) and their material appeared in the largest number of treatments (143), and members of the team contributed to a substantial number of additional records. The 1,487 specimens that had the elevation specified were collected below 2,000 m, mostly either between 500-1,000 or 1,500-2,000. Most specimens of both sexes but especially females were collected in July (931, 48.8%), more than twice as many as the second month, June (385, 20.2%). All records from these 2013 and 2014 publications were collected during the present decade. Jeremy A. Miller was the only author with more than one article (40%, two), but Klemen Candek was lead author on the most treatments (48.1%, 323) and documenting the most specimens (47.2%, 1,604).

### Aggregating data from XML publishing and legacy markup

The 41 articles from both journals contain a total of 996 treatments (884 species treatments) based on 8,231 specimens (species treatments contain citations of 8,172 specimens). About 3/4 of the treatments are published in Biodiversity Data Journal, but more than 58% of the specimens are cited among the much larger total number of Zootaxa articles (Fig. 5​). These charts (along with the charts on author and species, below) demonstrate the capability to combine data elements regardless of whether the semantic encoding is applied prospectively as part of an XML-based publication process or retrospectively through markup of legacy literature. ​

### Institutional collection: CAS

**Figure 6.**
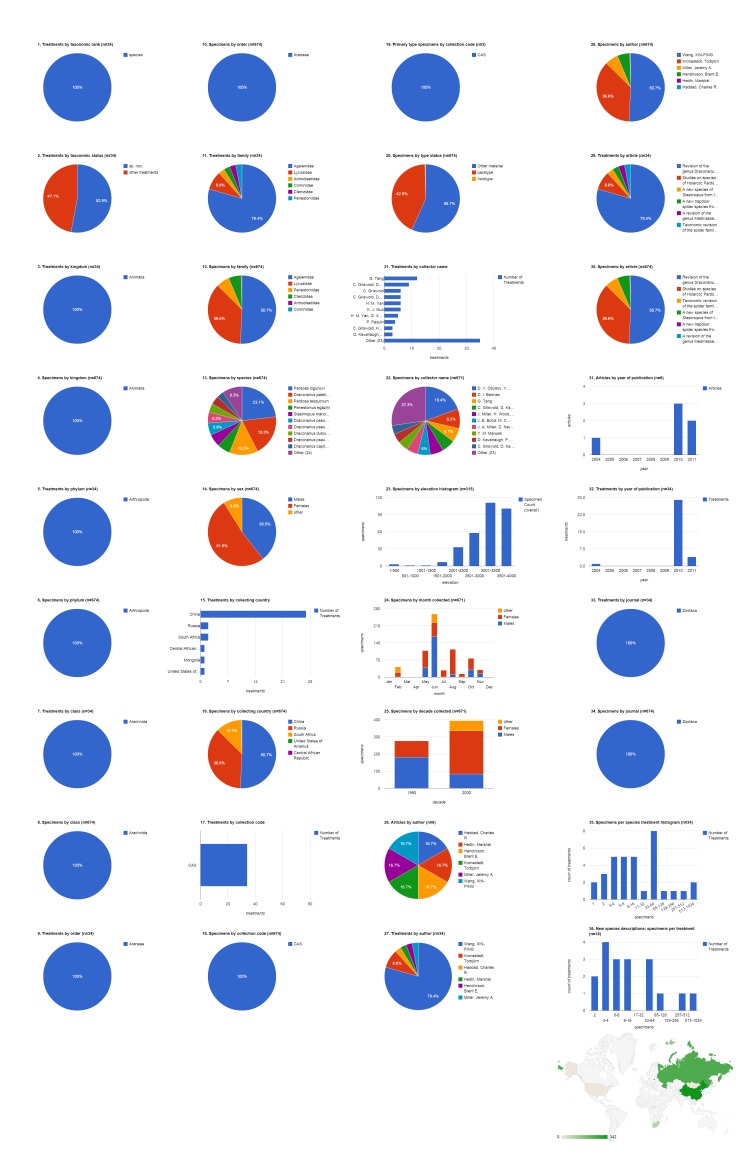
Dashboard charts summarizing content from species-rank treatments published in open access articles in Zootaxa and Biodiversity Data Journal containing treatments on spiders, filtered to show only specimens from the collection of the California Academy of Sciences (Suppl. material 10​). CAS was the institution associated with the largest number of specimens in this body of literature. ​All content shown here is from treatments published in Zootaxa; no CAS specimens were cited in Biodiversity Data Journal treatments on spiders.

The California Academy of Sciences collection contributed specimens to 34 treatments in six articles, all published in Zootaxa; most of them (29 treatments) in three articles published in 2010 (Fig. 6​). Most treatments concerned the family Agelenidae (79.4%, 27). Eighteen (52.9%) of the treatments were new species, but only three holotype specimens cited were archived in the CAS collection. Nearly all of the 688 specimens cited came from three countries: China (50.7%, 342), Russia (36.6%, 247), and South Africa (12.2%, 82). Specimens collected in China appeared in more treatments by far (27) than CAS specimens from any other country. The collecting team of D. V. Obydov and Y. M. Marusik led among collectors of cited CAS specimens (19.4%, 130). The 315 specimens that had the elevation specified were collected up to 4,000 m with the majority collected above 3,000 m. June was the peak month for cited CAS specimens, and all were collected in the 1990s or 2000s.

### Collecting country: Russia

**Figure 7.**
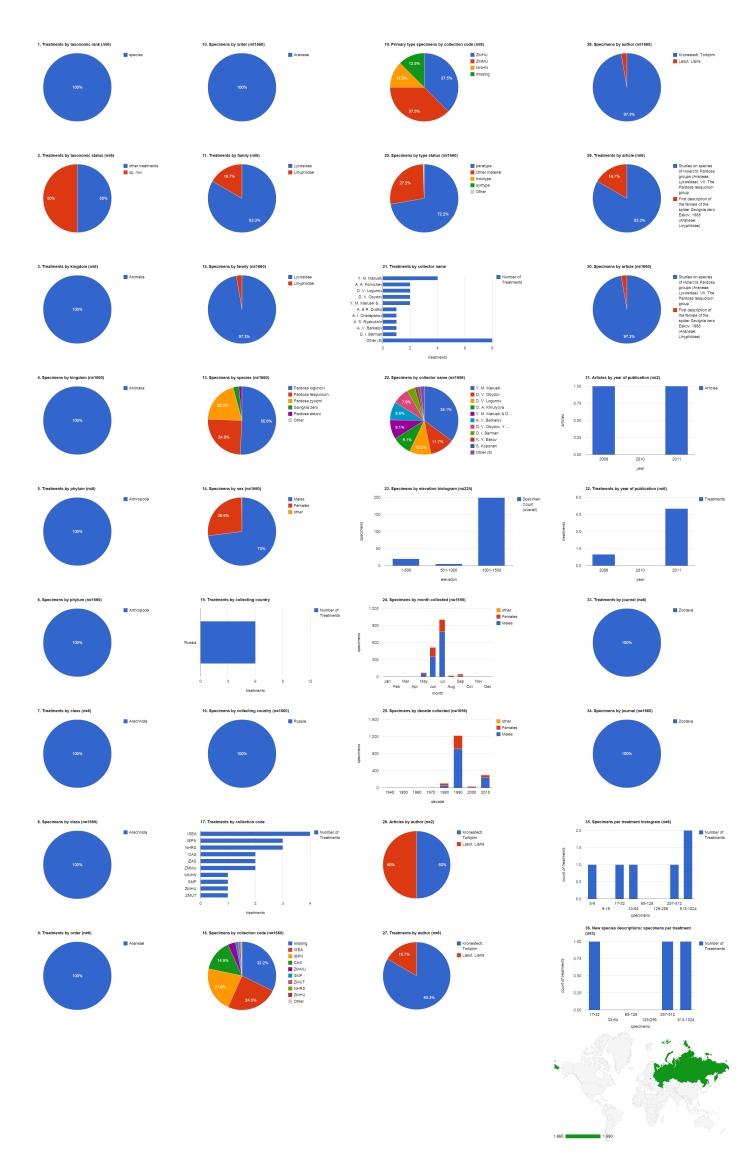
Dashboard charts summarizing content from species-rank treatments published in open access articles in Zootaxa and Biodiversity Data Journal containing treatments on spiders, filtered to show only specimens collected in Russia (Suppl. material 11​). Russia was the country associated with the largest number of specimens in this body of literature. ​​​All content shown here is from treatments published in Zootaxa; no specimens collected in Russia were cited in Biodiversity Data Journal treatments on spiders.

The 1,660 specimens collected in Russia were cited in six species treatments (five Lycosidae and one Linyphiidae), included three new species, and published in two articles in 2009 and 2011, both in Zootaxa (Fig. 7​). Adult males made up 73% (1,212) of the specimens, with the remainder divided between adult females (26.8%, 445) and non-adults (0.2%, three). Not counting the 32.2% (535) of specimens not explicitly associated in the source documents with an institutional collection, the largest repository for these specimens is the Institute for Systematics and Ecology of Animals, Novosibirsk, Russia (ISEA; 24.5%, 406). The ISEA had their specimens represented in the largest number of treatments (four). However, the Zoological Museum of Turku University, Finland (ZMHU) and the Zoological Museum of Moscow State University, Russia (ZMMU) tie for the largest number of primary type specimens from Russia cited (37.5%, three each). Yuri Marusik was the most productive specimen collector, alone collecting 581 (35.1%) specimens, not counting the specimens he collected collaboratively with others, and contributing material to the largest number of treatments (four). Most of the 224 specimens that had the collecting elevation specified were collected between 1,001-1,500 m. Most specimens of both sexes but especially males were collected in July, followed by June. The bulk of the specimens cited in this group of publications were collected in the 1990s.

### Collector name: Y. M. Marusik

**Figure 8.**
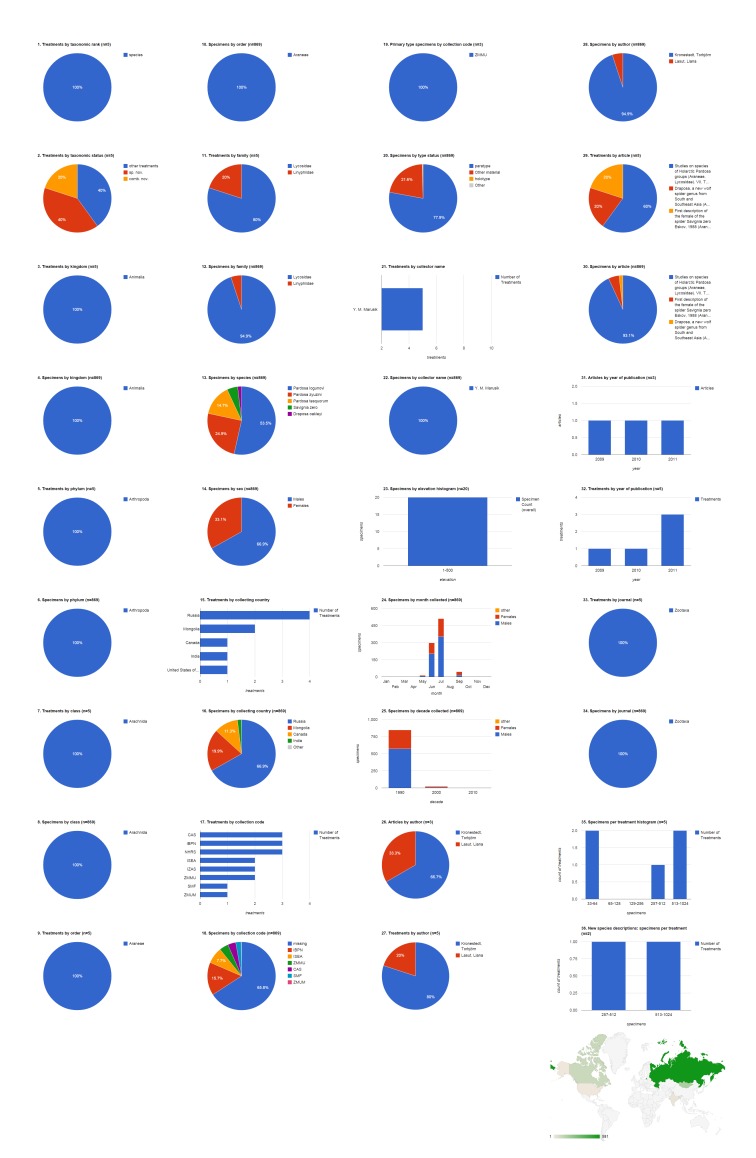
Dashboard charts summarizing content from species-rank treatments published in open access articles in Zootaxa and Biodiversity Data Journal containing treatments on spiders, filtered to show only specimens collected by Y. M. Marusik (Suppl. material 12​). Marusik was the collector associated with the largest number of specimens in this body of literature. Note that this count excludes specimens that Marusik collected collaboratively with others (see Discussion: Tracking Individuals). ​​​All content shown here is from articles published in Zootaxa; no specimens collected by Marusik were cited in Biodiversity Data Journal treatments on spiders.

The 869 specimens collected solely by Y. M. Marusik were cited in five treatments (four Lycosidae and one Linyphiidae) including two new species, and were published in three articles, all appearing in Zootaxa (Fig. 8​). Most specimens (66.9%, 581) were collected in Russia. The specimens that Marusik collected in Russia contributed to four treatments. Most of the specimens were males (66.9%, 581) collected in June or July during the 1990s. Only one collection of 20 specimens had the elevation specified in the source publication. Not counting the 65.8% (572) of specimens not explicitly associated in the source documents with an institutional collection, the largest repository for these specimens is the Institute for Biological Problems of the North, Magadan, Russia (IBPN; 15.7%, 136). The three primary type specimens collected by Marusik are deposited in the Zoological Museum of Moscow State University, Moscow, Russia (ZMMU).

### Article: Kronestedt and Marusik 2011

This article is the seventh in a series on Holarctic members of the wolf spider genus *Pardosa* (Lycosidae). The publication contains seven species treatments and one higher (species group) treatment based on 1,957 specimens in the family Lycosidae; three of the treatments are new species (Fig. 9a​). Adult males made up 70% (1,370) of the specimens, with the remainder divided between adult females (29.8%, 583) and non-adults (0.2%, 4). Specimens cited in this article are mostly from Russia (82.5%, 1,615), with Mongolia (9%, 176), Canada (7.3%, 142), China (0.9%, 17), and the United States of America (0.4%, 7) also represented; five of the treatments include specimens from Russia. Not counting the 35.5% (695) of specimens not explicitly associated with an institutional collection, the largest repository for these specimens is the Institute for Biological Problems of the North, Magadan, Russia (IBPN; 23.7%, 464). Four of the 12 primary type specimens cited were archived in the Zoological Museum of Moscow State University, Russia (ZMMU). Specimens cited in five treatments are deposited at both NHRS (Swedish Museum of Natural History, Stockholm, Sweden) and IZAS (Institute of Zoology, Chinese Academy of Sciences, Beijing, China). Nearly all of the 204 specimens for which the collecting elevation was specified were taken from 1,001-1,500 m. Y. M. Marusik was by far the most productive specimen collector, alone collecting 809 (41.7%) specimens, not counting the specimens he collected collaboratively with others. Most specimens of both sexes, but especially males, were collected in July, followed by June. The bulk of the specimens cited in this article were collected in the 1990s. Three treatments are based on hundreds of specimens; four treatments are based on no more than a few dozen specimens, but none is based on a singleton.

### Treatment: *Pardosa zyuzini* in Kronestedt & Marusik 2011

*Pardosa zyuzini* is one of seven species treatments and one of three new species in Kronestedt and Marusik (2011). 25.8% (504) of the specimens in the article are attributed to this species (Fig. 9b​). Adult males made up 71.4% (360) of the specimens, with the remainder being adult females (28.6%, 144). Specimens were collected in Russia (66.5%, 335) and Mongolia (33.5%, 169). The largest repository for these specimens is the Institute for Biological Problems of the North, Magadan, Russia (IBPN; 39.1%, 197). The holotype is deposited in the Zoological Museum of Moscow State University, Russia (ZMMU). Yuri Marusik was by far the most productive specimen collector, alone collecting 216 (42.9%) specimens, not counting the specimens he collected collaboratively with others. Nearly all of the 204 specimens for which the collecting elevation was specified were taken from 1,001-1,500 m. Most specimens were collected in July, but the largest number of females were found in June. Most of the specimens were collected during the 1990s.

### Species: *Tenuiphantes tenuis*

**Figure 10.**
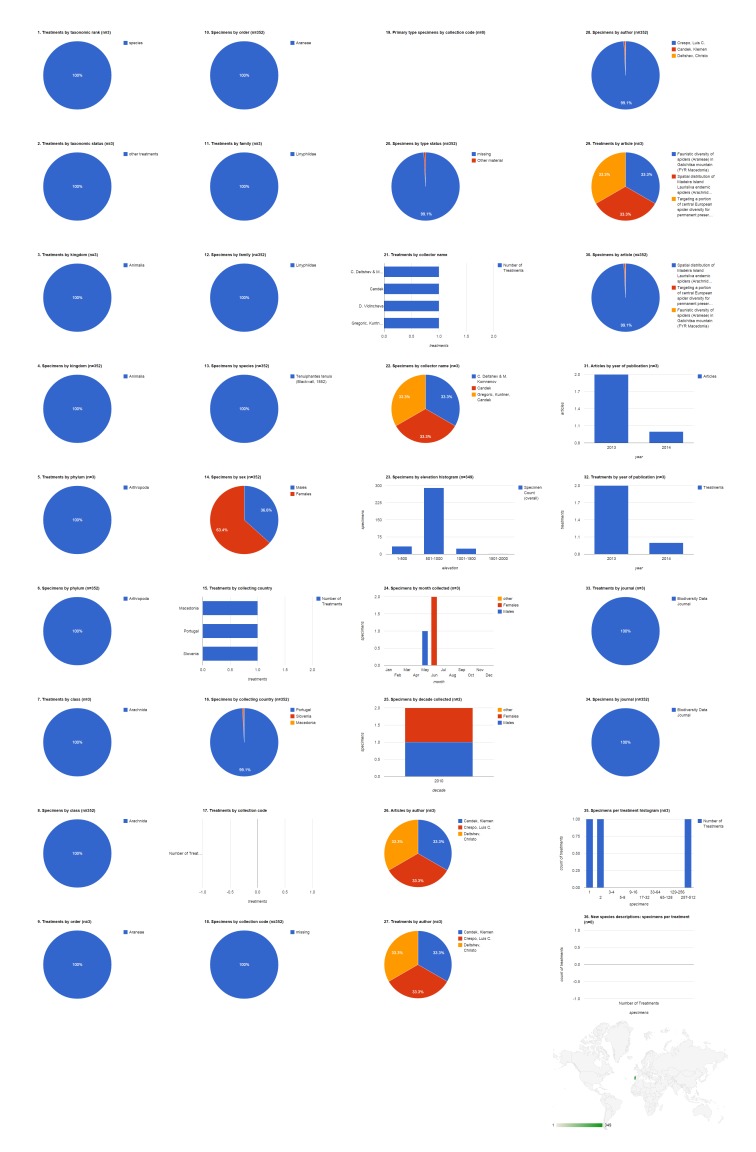
Dashboard charts summarizing data published in open access articles in Zootaxa and Biodiversity Data Journal containing treatments on spiders, filtered to show only one species: *Tenuiphantes tenuis* (Suppl. material 15​). Data on *Tenuiphantes tenuis* was included in three treatments, all published in Biodiversity Data Journal.

Records of this linyphiid spider based on a total of 352 specimens appeared in three articles, all published in Biodiversity Data Journal (Fig. 10​​). Nearly all of these were from the Portuguese island of Madeira with the remaining records from Macedonia and Slovenia. Most of the specimens were female (63.4%, 223). None of these records explicitly cited a collection in their structured text, although some cite a depository in the body of the article (see Discussion). Collector name, collecting date, and type status were also omitted from most of the structured text.

### Author: Jeremy A. Miller

**Figure 11.**
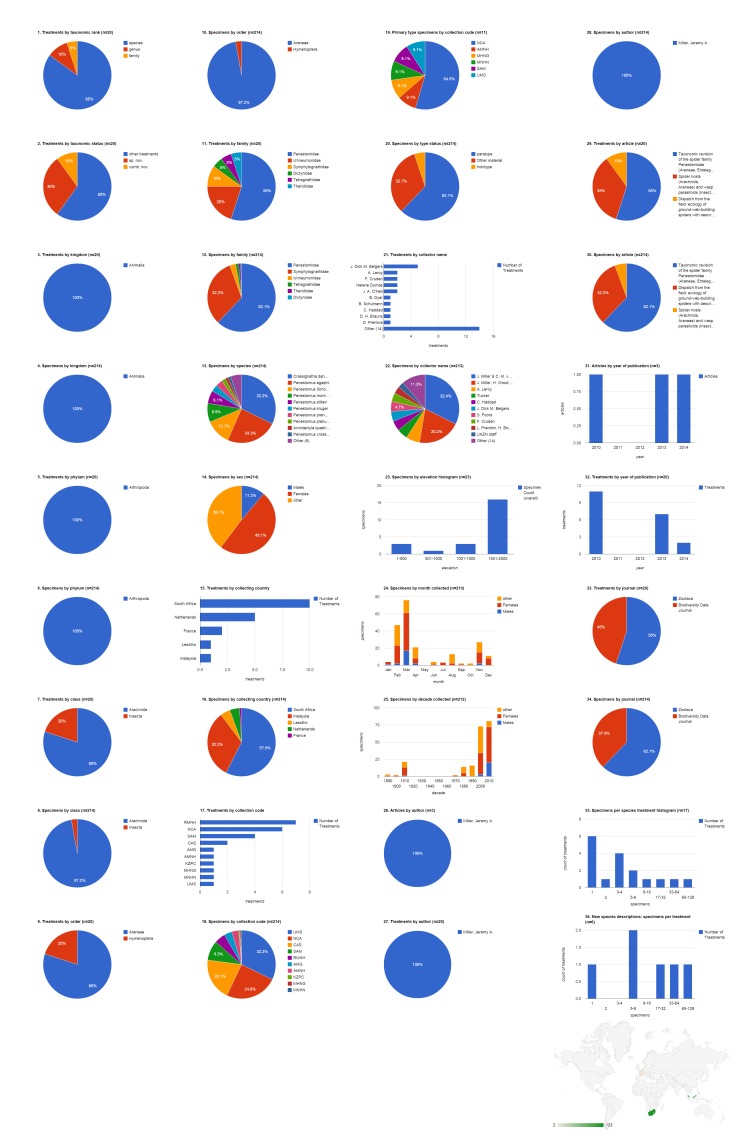
Dashboard charts summarizing data published in open access articles in Zootaxa and Biodiversity Data Journal containing treatments on spiders, filtered to show only one lead author: Jeremy A. Miller (Suppl. material 16​). Miller was lead author on two publications in Biodiversity Data Journal and one in Zootaxa, and was the only lead author on open access publications featuring spider treatments in both journals. Note that in addition to the three articles on which he is lead author, he is also a contributing author to a fourth article (Wang et al. 2010), but content from this article is not included here (see Discussion: Tracking individuals).

Jeremy A. Miller was the lead author on two publications in Biodiversity Data Journal and one open access publication in Zootaxa containing treatments on spiders (​Fig. 11​). Collectively, these articles contain 20 treatments (17 species rank) including 6 new species and 2 new combinations. Four of these treatments concern Hymenoptera (Ichneumonidae, 6 specimens), the remainder concern the spider families Penestomidae, Symphytognathidae, Dictynidae, Tetragnathidae, and Theridiidae, based on a total of 208 specimens. Six treatments, including one new species, were based on singletons. Most specimens were collected in South Africa (57.5%, 123), followed by Malaysia (32.2%, 69). The specimen sex ratio exhibited a pronounced female bias, accounting for 105 of 129 sexually mature specimens; an additional 85 specimens recorded were not sexually mature. The largest number of specimens (32.2%, 69) were deposited in Universiti Malaysia Sabah's Institute for Tropical Biology and Conservation, Borneensis (UMS), but the largest number of primary type specimens cited (54.5%, six) were deposited in South Africa's National Collection of Arachnida in Pretoria (NCA). The largest number of specimens was collected during March, followed by February. Most of the specimens were collected since 2,000, but a modest number were collected during the 1910s and a few as far back as the 1890s. Only 23 specimens had associated elevation data, but of those, most were collected between 1,500-2,000 m.

## Discussion

### Data interoperability and prospective publishing: recommendations for BDJ authors

Specimen data associated with spiders (among other taxa) are often structured to reflect multiple individual specimens in a single vial. A lot of specimens may include adult males, adult females, and sexually immature specimens for which sex is undetermined; all of these specimens may be associated with a single specimen code. We find that abundance and sex capture useful information about specimen data. For example, the phenology of males and females may be different, so the ability to determine what time of year a particular sex has been collected can be valuable to an investigator planning field work. Darwin Core expects a lot of specimens to all be of the same type (e.g., sex) and does not offer a simple way to represent heterogeneous objects in a single record (see https://github.com/tdwg/dwc/issues/35, https://github.com/tdwg/dwc/issues/36). To work around this, Plazi's SRS can parse the number of male and female specimens entered in the "sex" field (e.g., 1 male, 4 females), and either sum these values to determine the total abundance or use the individualCount field for this purpose (the individualCount can be higher than the sum of males and females when non-adult specimens are present; the difference between individualCount and the sum of males and females is automatically added to the category "other"). We recommend that authors of taxonomic treatments in BDJ complete both the individualCount field and sex field with their materials citations.

Information on the institution where particular specimens were deposited was frequently absent from the structured materials citations data in BDJ articles (Fig. 4​). Even when the institution is specified in the body of the article, that information becomes disassociated from the digital records themselves as they become disseminated to cybertaxonomic resources, such as Plazi and GBIF. We therefore recommend that authors include this information with their structured records. Similarly, collector name, collecting date, and type status should be included with records whenever possible.

Biodiversity Data Journal was launched with the motto: "making small data big." Realizing the potential of this vision requires that authors contribute structured specimen data with a sufficient level of granularity and detail. Otherwise, the power of data aggregation is curtailed.

### Legacy data: materials citations with ambiguous structure

**Figure 12.**
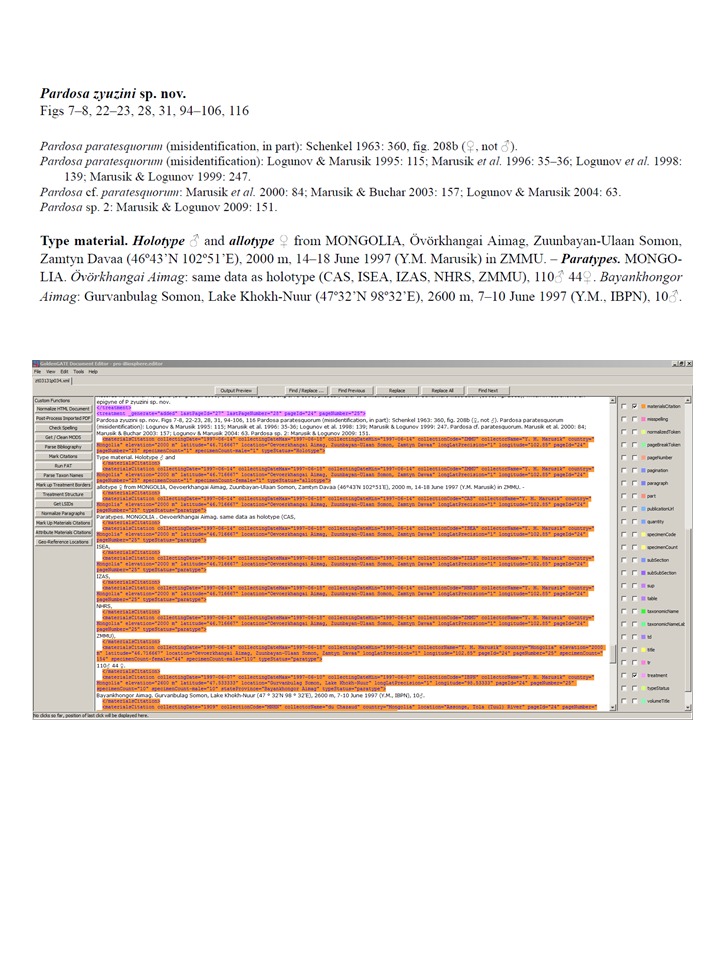
Excerpt from a taxonomic treatment with ambiguously structured materialsCitations data in the source document. The top frame shows the published PDF, the lower frame shows the same content in GoldenGATE with the treatment and materialsCitation tags revealed. The source document is ambiguous about how many paratype specimens are deposited in which natural history collection. This is represented in XML by associating the collection event data (place, time, collector) with each of the listed institutional collections but no quantity of specimens is assigned to any collection. The 110 male and 44 female specimens are also associated with the collection event data, but with no institutional collection.

The extent to which primary specimen data can be extracted from legacy literature depends on how the data are structured. Ideally, the data that refer to 'materials citations' link particular specimens to particular institutions and collecting events. Occasionally, specimen counts, institutions, and collecting events are disassociated. For example, in the treatment on *Pardosa zyuzini* (Kronestedt and Marusik 2011; Fig. 12), 154 paratype specimens are cited from the type locality in Mongolia and are distributed among five institutions. Exactly how many specimens are in which institution is not reported. From such ambiguously structured text, we can count the number of specimens reported for this species, and deduce that specimens from particular localities are present in each of the specified institutions, but we cannot count the number of specimens in each institution. Such records appear in the “Specimens by collection code” chart as “missing” (note that the term collectionCode as used by Plazi is equivilent to institutionCode in Darwin Core; this unfortunately confusing termanology should be reconciled in the future). In the XML file, the locality and collecting event data are associated with each of the five institutions (collectionCode), and the specimen count data are associated with the locality and collecting event, but no collectionCode.​

### Legacy data: incomplete integration with GBIF

XML structured documents in Plazi are available for aggregation by GBIF. The treatments from Zootaxa included in this study supply records for 120 species that otherwise have no records in GBIF. However, the records for 98 of these species are not yet visible on GBIF. In most cases, the reason appears to be that the species is not in the GBIF backbone taxonomy (Döring 2015​). This means that more than half of the treatments marked up for this study contributed structured data for species that otherwise had no such data available. The fact that most of these data are not currently available for aggregation and reanalysis on GBIF is unfortunate and in need of attention. This project concerns recent legacy literature and while these names have been incorporated into the World Spider Catalog (http://www.wsc.nmbe.ch/), the catalog has only recently been restructured in a way that facilitates synchronization with online resources such as GBIF; this has not yet been implemented. It may be that this problem will rectify itself in due course. Alternatively, with the development of appropriate mechanisms, Plazi could contribute taxonomic names directly to GBIF or to the Catalogue of Life, which provides taxonomic data to GBIF and other online biodiversity databases.

### Tracking people

One limitation of the way specimen data are currently structured is that the "collectorName" field does not parse out individual collectors when more than one person is involved. The problem that this creates is evident in the case of the most prolific collector. Marusik collected specimens alone and in collaboration with others, including D. V. Obydov. Some records listed Marusik as the first collector, others listed Obydov first. These are counted in the “Specimens by collector name” chart as three different collectors (1: Y. M. Marusik, 2: Y. M. Marusik & D. V. Obydov, 3: D. V. Obydov & Y. M. Marusik). In such cases, we may wish to attribute half of the specimens to each collector (or more generally, divide the number of specimens collected by the number of collectors and attribute that fraction to each member of the team). Alternatively, we might want to count the number of specimens that each individual contributed to collecting, which would result in a total count of specimens equal to or greater than the actual number collected. Similarly, we see in the “Treatments by collector name” chart for all data that Charles Griswold appears, alone or with collaborators, in four of the top ten rankings. A search of the underlying data reveals that Griswold, alone or with collaborators, contributed specimens to 22 treatments. As currently structured, it is not easy to track this.

In addition to specimen collector, individuals have two other main roles in taxonomic research: as publication (co-)authors and as taxonomic authorities (in which case the name of one or more individuals is associated with a taxonomic name). Both of these can be conducted as solo or collaborative activities. Summaries of individual contributions to taxonomic research should allow us to derive data on all three functions so that scientists get due credit for their efforts.

### Data exchange and research metrics

Data exchange between taxonomic research and institutional collections is less sophisticated currently than it could be. Collections-based institutions have an interest in monitoring how their collections are used in taxonomic research. XML-structured documents make it possible to import data from marked up literature into collections databases, making such databases more current and complete. Institutions can be compared by the number of specimens cited, or the number of treatments contributed to. Such metrics could produce healthy competition among institutions, stimulate specimen circulation in support of taxonomic research, and provide quantitative metrics in support of further funding. Similar metrics could form a basis for nuanced comparison of individual researchers. We might be interested not just in the number of treatments published, but in the number of specimens examined per treatment. We might be interested in the number of specimens and taxa collected by an individual that appear in published taxonomic research. Instead of looking just at individuals, these data could be aggregated by categories such as institution or country.

As more structured data from legacy literature become available and are shared with collections databases, it will become necessary to recognize instances of the same object cited in a database and one or more publications. Taxonomic research typically proceeds through the re-examination of specimens previously studied and cited by other taxonomists, including but not limited to primary type specimens. Without a mechanism to identify these as the same object, the total number of specimens known could become difficult to discern. Likewise, the same specimen recorded in a collections database and cited by a taxonomic paper should be recognized as one object, not two. This highlights the need for globally unique identifiers for specimens, whose application remains problematic in biodiversity informatics (Guralnick et al. 2014, Guralnick et al. 2015). Only 40% of the records investigated for this study (1,144 of 2,894, representing 26% of the specimens, 2,156 of 8,231) had some form of specimen code reported in the literature. Instiututionally unique identifiers can suggest that two matching records refer to the same object, but a robust global system for tracking specimens and their associated data and usage history remains an elusive challenge.

### Research questions and taxonomic literature

Some recent publications have used specimen data from taxonomic literature to investigate aspects of biodiversity knowledge. We adapt our spider dataset to two of these questions: 1) the time lag between species discovery and description, and 2) the prevalence of rare species in species descriptions.

Fontaine et al. (2012) investigated the pace of biodiversity description based on a sample of species described in 2007. Applying the same approach to this spider dataset, we find that the mean delay between the first collection and description of the 92 new species in the data set is 17.2 years (standard error: 2.37) with a median of seven years, ranging between 102 (the lycosid *Pardosa zyuzini* Kronestedt & Marusik 2011) and zero years (i.e., published the same year it was discovered; the symphytognathid *Crassignatha danaugirangensis* Miller et al. 2014). Fontaine et al. did not break out data on spiders separately, but our values fit closely with their “other invertebrates” category. This means that the average shelf life between discovery and description for spiders is about four years less than for insects, and about 15 or more years less than for vertebrates and plants.

Lim et al. (2011) surveyed taxonomic literature to investigate the prevalence of rare species. Rare species are a conspicuous part of community structure, particularly prevalent among tropical arthropods (Coddington et al. 2009, Novotný and Basset 2000). Lim et al. (2011) found that rare species are prevalent in the taxonomic literature. Rare species include singletons (taxa known from a single specimen) and uniques (taxa known from a single collection event). Based on 25 articles on an assortment of arthropod taxa (including spiders) published in the monograph series of the American Museum of Natural History (2000-2010), 17.7% of new species are described from a single specimen. The Zootaxa spider dataset describes new species from singletons at more than twice this rate (34/91 = 37.4%). Another way of looking at rarity is by looking at the number of localities a new species has been found in (the incidence-based as opposed to the abundance-based approach). The American Museum of Natural History invertebrate data indicated that 27.5% of new species are described from a single locality; the Zootaxa spider data were again richer in rarity with 38.5% (35/91).

A key difference between these previous efforts and our literature markup project is that the primary data upon which our analyses are based are readily accessible to all from Plazi (http://plazi.cs.umb.edu/GgServer/srsStats) and can be built upon as treatments are added. Meier and Dikow (2004) and Dikow et al. (2009) presented several ideas for how specimen data from taxonomic revisions could be applied to biodiversity research. These include comparing areas for conservation prioritization, data-driven assessment of species conservation status, and predicting the number of species in a group yet to be described. While their ideas call for denser coverage of geographic areas than permitted by our dataset, their work continues to serve as a guidepost to what the future of XML marked taxonomic literature could achieve.

### Prospective taxonomic publishing: the end of unstructured taxonomic literature

The rate of publication in taxonomy continues to rise, as does the potential for accelerated transition from discovery to publication (Miller et al. 2014). Therefore, while addressing the challenge of legacy literature XML markup, we should not continue adding to the backlog. There have been great recent advances in taxonomic publishing. Pensoft (http://www.pensoft.net/about.php​) journals have led the way with an XML-based approach that facilitates the reuse, aggregation, and dissemination of content to an increasing variety of cybertaxonomic databases and resources. Whether integral to the publication process or added later, structured taxonomic treatments promote transparency and repeatability in biodiversity science while facilitating the aggregation and reanalysis of data.

### Conclusions

The modest pilot demonstration presented here is focused on spider literature, but the approach can be applied to any taxonomic literature. Our accumulated biodiversity knowledge includes an estimated 2-3 billion specimens in natural history collections and 500 million pages of printed text (Thessen and Patterson 2011, Ariño 2010​). These are the data we need to answer questions that are relevant to our world today, like setting conservation priorities and anticipating the effects of climate change on biodiversity and ecosystem functions that affect the lives of people. Computer models of the biosphere are becoming increasingly sophisticated and powerful, and this field appears to have tremendous potential for growth in the near future (Purves et al. 2013). To fully realize the benefits of these nascent technological advances, we are going to have to wrestle with the gap between the knowledge we have accumulated in libraries over 250 years of research and the data that are available in structured digital form so they can be used by computers. In short, we have half a billion pages worth of biodiversity knowledge and no way to query it. XML tagging, whether applied to prospective publications as in Biodiversity Data Journal or to legacy publications using tools like GoldenGATE, makes the primary data available in digital structured form. This enhances the scientific quality of the work because it is easier for other scientists to re-evaluate and test conclusions based on the data. But the real power comes when data from many articles are combined, queried, and reused for new purposes. Potential applications for these data span the scientific, policy, and public spheres (Arzberger et al. 2004, Hardisty et al. 2013​). When we all have better access to the information that already exists in the global biodiversity library, this helps us do a better job of exploring what we don’t know and wisely applying what we do.

## Supplementary Material

Supplementary material 1 Global Biodiversity Information Facility: Taxa and Records Data type: Data exported from GBIF, zip archive contains one csv file with raw data and one xlsx file with data summary
Brief description: All records in GBIF with taxonomic ranks (kingdom, phylum, class, order, and species), basis of record (e.g., preserved specimen), and count of records, exported from GBIF on 7 December 2014.
File: oo_41611.zip GBIF

Supplementary material 2 World Spider Catalog Bibliographic Data: Publications Data type: Count of publications by journal/publisher
Brief description: Ranked list of journal/publisher exported from the World Spider Catalog 14 October 2014 with total articles by source, cumulative articles, and cucmulative proportion of articles.
File: oo_41595.csv World Spider Catalog

Supplementary material 3 World Spider Catalog Bibliographic Data: Treatments Data type: Count of treatments by journal/publisher
Brief description: List of journal/publisher by ranked by treatment count exported from the World Spider Catalog 14 October 2014 with total treatments by source, cumulative treatments, and cumulative proportion of treatments.
File: oo_41597.csv World Spider Catalog

Supplementary material 4 Legacy literature dashboard: all treatments Data type: HTML/Javascript
Brief description: Dashboard charts summarizing content from 37 open access articles published in Zootaxa containing treatments on spiders. This page shows data from all treatments. When viewed using a browser (such as Google Chrome) with an internet connection, this page sends a series of queries to Plazi and integrates the results with the Google Charts API to produce 37 dashboard charts.
File: oo_41613.html Miller et al.

Supplementary material 5 Legacy literature dashboard: species-rank treatments Data type: HTML/Javascript
Brief description: Dashboard charts summarizing content from 37 open access articles published in Zootaxa containing treatments on spiders. This page shows data from species-rank treatments. When viewed using a browser (such as Google Chrome) with an internet connection, this page sends a series of queries to Plazi and integrates the results with the Google Charts API to produce 37 dashboard charts.
File: oo_41614.html Miller et al.

Supplementary material 6 Prospective publishing dashboard: all treatments Data type: HTML/Javascript
Brief description: Dashboard charts summarizing content from 5 articles published in Biodiversity Data Journal containing treatments on spiders. This page shows data from all treatments. When viewed using a browser (such as Google Chrome) with an internet connection, this page sends a series of queries to Plazi and integrates the results with the Google Charts API to produce 37 dashboard charts.
File: oo_41615.html Miller et al.

Supplementary material 7 Prospective publishing dashboard: species-rank treatments Data type: HTML/Javascript
Brief description: Dashboard charts summarizing content from 5 articles published in Biodiversity Data Journal containing treatments on spiders. This page shows data from species-rank treatments. When viewed using a browser (such as Google Chrome) with an internet connection, this page sends a series of queries to Plazi and integrates the results with the Google Charts API to produce 37 dashboard charts.
File: oo_41616.html Miller et al.

Supplementary material 8 Integrated legacy literature and prospective publishing dashboard: all treatments Data type: HTML/Javascript
Brief description: Dashboard charts summarizing content from 42 articles published either as open access articles published in Zootaxa or in Biodiversity Data Journal, containing treatments on spiders. This page shows data from all treatments. When viewed using a browser (such as Google Chrome) with an internet connection, this page sends a series of queries to Plazi and integrates the results with the Google Charts API to produce 37 interactive dashboard charts.
File: oo_41621.html Miller et al.

Supplementary material 9 Integrated legacy literature and prospective publishing dashboard: species-rank treatments Data type: HTML/Javascript
Brief description: Dashboard charts summarizing content from 42 articles published either as open access articles published in Zootaxa or in Biodiversity Data Journal, containing treatments on spiders. This page shows data from species-rank treatments. When viewed using a browser (such as Google Chrome) with an internet connection, this page sends a series of queries to Plazi and integrates the results with the Google Charts API to produce 37 interactive dashboard charts.
File: oo_41622.html Miller et al.

Supplementary material 10 Institutional collection dashboard: specimens from the collection of the California Academy of Sciences (CAS) Data type: HTML/Javascript
Brief description: Dashboard charts showing only specimens from the collection of the California Academy of Sciences. This page shows data from species-rank treatments. When viewed using a browser (such as Google Chrome) with an internet connection, this page sends a series of queries to Plazi and integrates the results with the Google Charts API to produce 37 interactive dashboard charts.
File: oo_41635.html Miller et al.

Supplementary material 11 Collecting country dashboard: specimens collected in Russia Data type: HTML/Javascript
Brief description: Dashboard charts showing only specimens collected in Russia. This page shows data from species-rank treatments. When viewed using a browser (such as Google Chrome) with an internet connection, this page sends a series of queries to Plazi and integrates the results with the Google Charts API to produce 37 interactive dashboard charts.
File: oo_41636.html Miller et al.

Supplementary material 12 Collector dashboard: specimens collected by Y. M. Marusik Data type: HTML/Javascript
Brief description: Dashboard charts showing only specimens collected by Y. M. Marusik. This page shows data from species-rank treatments. When viewed using a browser (such as Google Chrome) with an internet connection, this page sends a series of queries to Plazi and integrates the results with the Google Charts API to produce 37 interactive dashboard charts.
File: oo_41637.html Miller et al.

Supplementary material 13 Article dashboard: content from Kronestedt and Marusik (2011) Data type: HTML/Javascript
Brief description: Dashboard charts showing content from one article, Kronestedt and Marusik 2011. This page shows data from all treatments. When viewed using a browser (such as Google Chrome) with an internet connection, this page sends a series of queries to Plazi and integrates the results with the Google Charts API to produce 37 interactive dashboard charts.
File: oo_41638.html Miller et al.

Supplementary material 14 Treatment dashboard: content from Pardosa zyuzini treatment in Kronestedt and Marusik (2011) Data type: HTML/Javascript
Brief description: Dashboard charts showing content from one treatment: Pardosa zyuzini in Kronestedt and Marusik (2011). When viewed using a browser (such as Google Chrome) with an internet connection, this page sends a series of queries to Plazi and integrates the results with the Google Charts API to produce 37 interactive dashboard charts.
File: oo_41639.html Miller et al.

Supplementary material 15 Species dashboard: Tenuiphantes tenuis Data type: HTML/Javascript
Brief description: Dashboard charts showing only specimens of the linyphiid spider Tenuiphantes tenuis. When viewed using a browser (such as Google Chrome) with an internet connection, this page sends a series of queries to Plazi and integrates the results with the Google Charts API to produce 37 interactive dashboard charts.
File: oo_41640.html Miller et al.

Supplementary material 16 Author dashboard: Jeremy A. Miller Data type: HTML/Javascript
Brief description: Dashboard charts showing content from articles by Jeremy A. Miller (lead author). When viewed using a browser (such as Google Chrome) with an internet connection, this page sends a series of queries to Plazi and integrates the results with the Google Charts API to produce 37 interactive dashboard charts.
File: oo_41641.html Miller et al.

Supplementary material 17 Generating Interactive Dashboard Charts Based on Plazi Treatment Data Data type: Microsoft Word document
Brief description: A brief explanation of how to create and customize interactive charts using data on Plazi.
File: oo_43194.docx Jeremy A. Miller et al.

## Figures and Tables

**Figure 1a.**
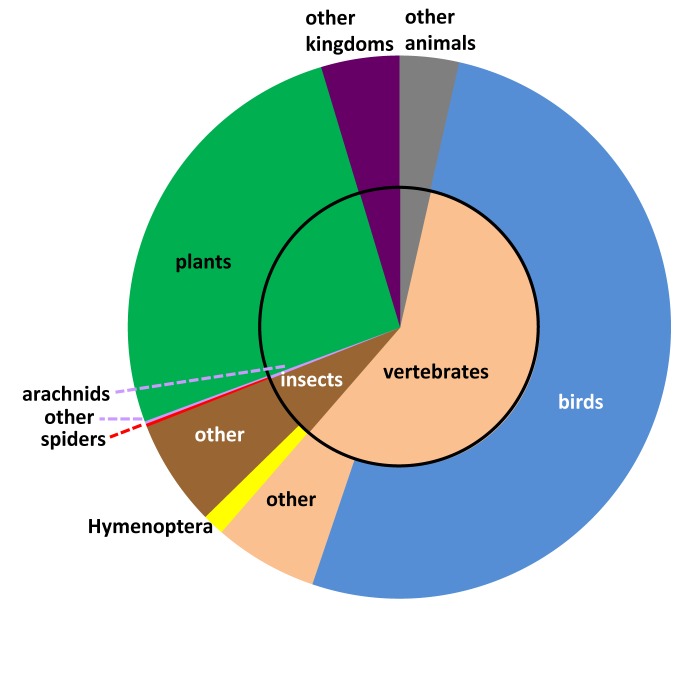
All records in GBIF (*n* = 517,325,595).

**Figure 1b.**
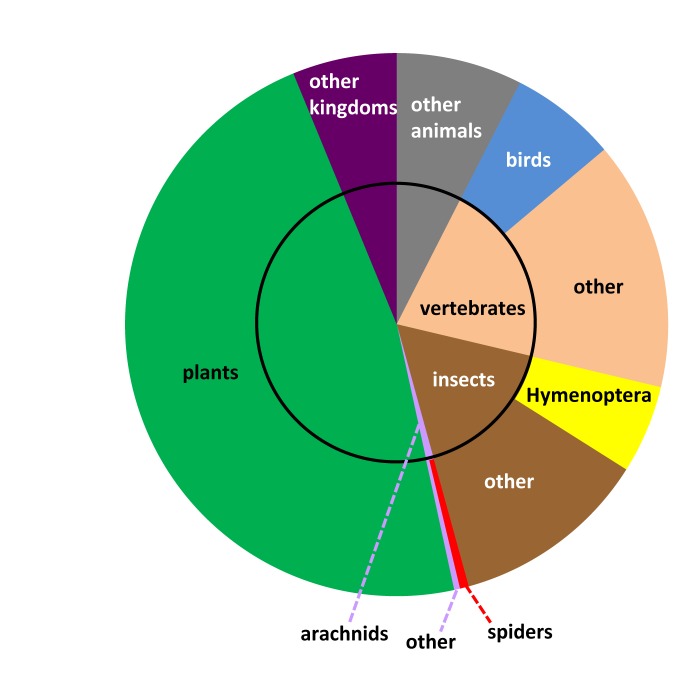
Specimen-based records in GBIF (*n* = 98,144,242).

**Figure 2a.**
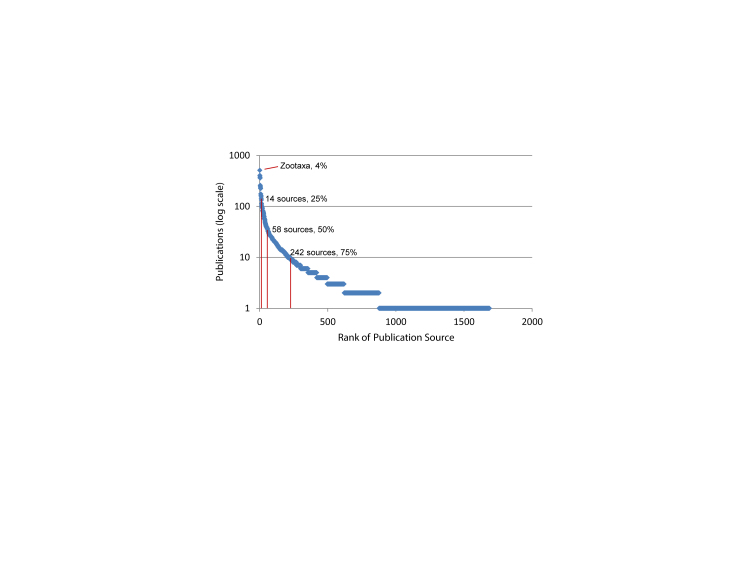
12,377 publications listed in the 2014 World Spider Catalog. Zootaxa is the top ranking venue with 509 titles representing just over 4% of spider taxonomy.

**Figure 2b.**
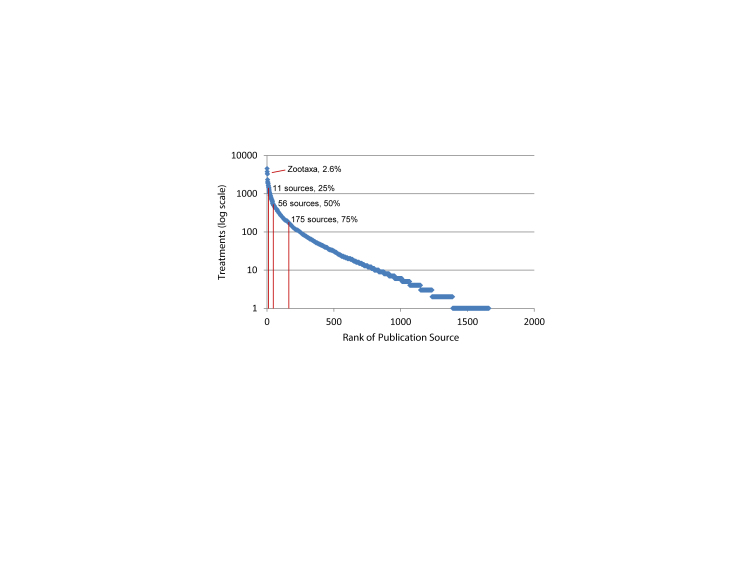
The 2014 World Spider Catalog refers to 126,621 treatments. With 3314 treatments (2.6%), Zootaxa is the third ranking all time venue behind two museum monograph series: Bulletin of the American Museum of Natural History (4537 treatments, 3.6%) and Harvard's Bulletin of the Museum of Comparative Zoology (3761 treatments, 3.0%).

**Figure 3a.**
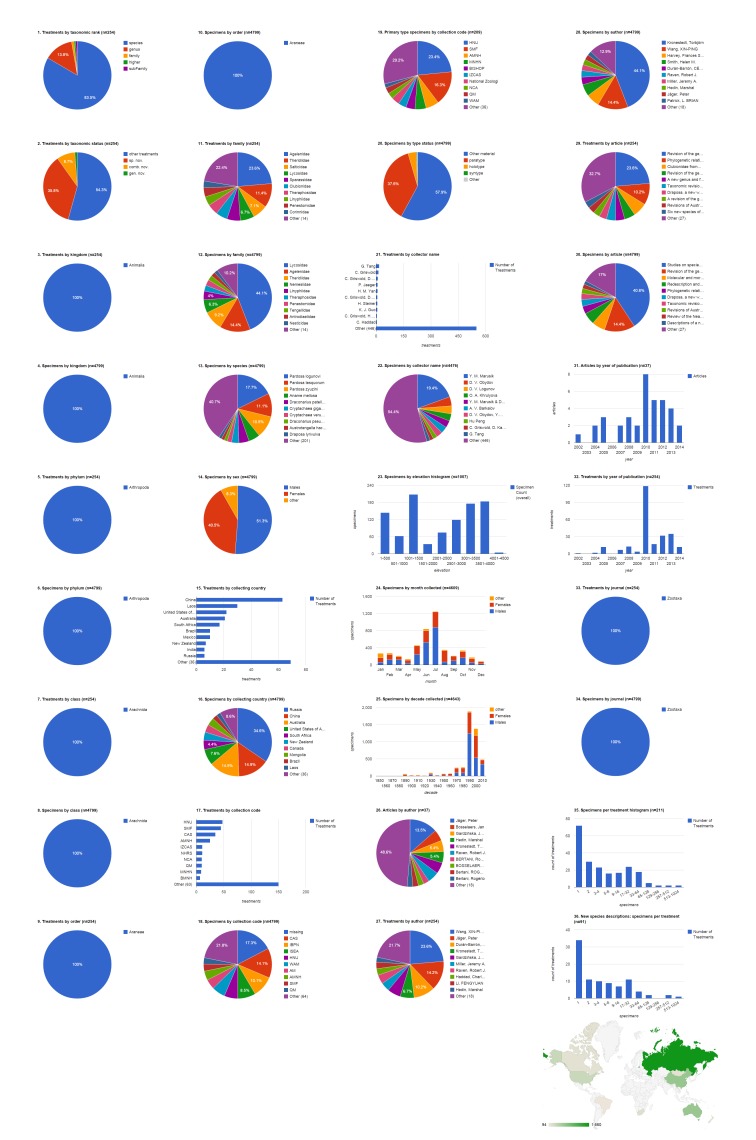
All treatments regardless of taxonomic rank.

**Figure 3b.**
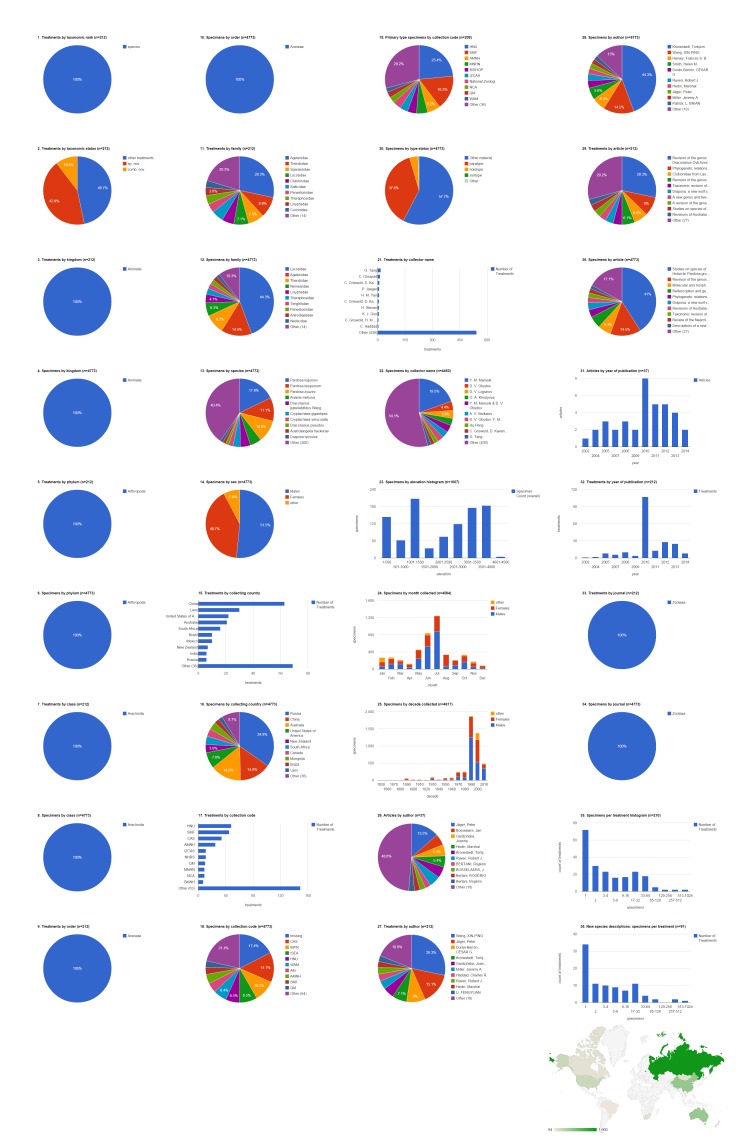
Species-rank treatments.

**Figure 4a.**
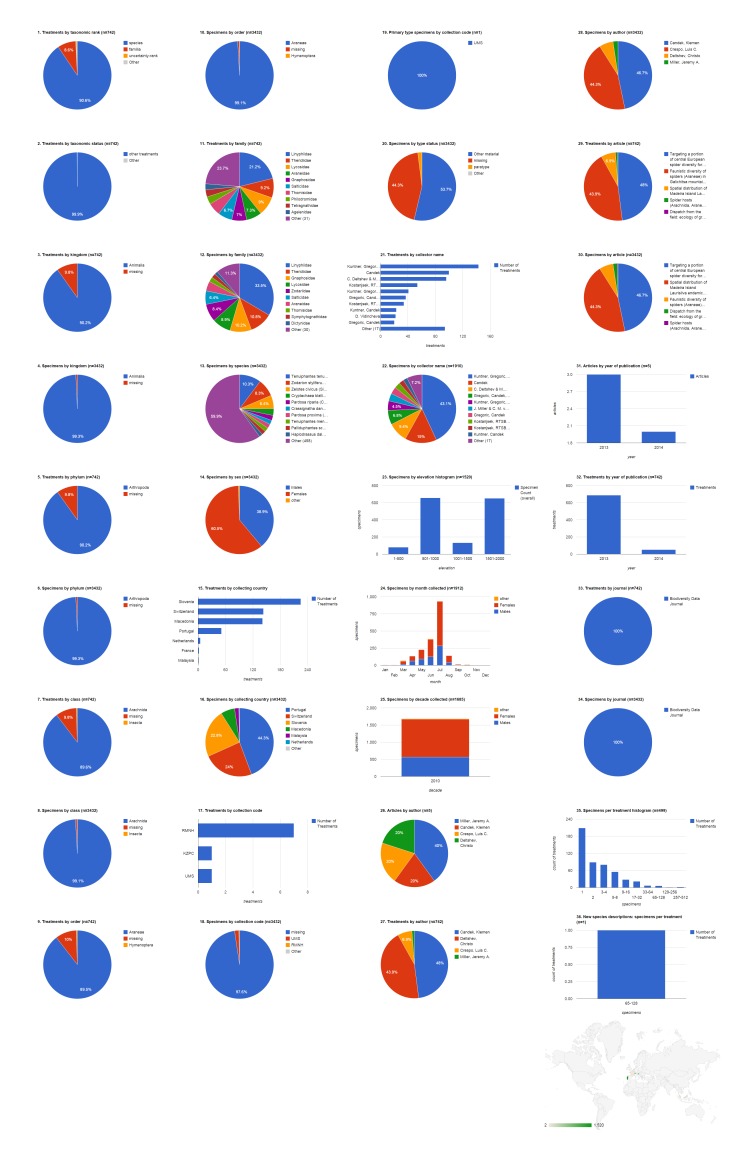
All treatments regardless of taxonomic rank.

**Figure 4b.**
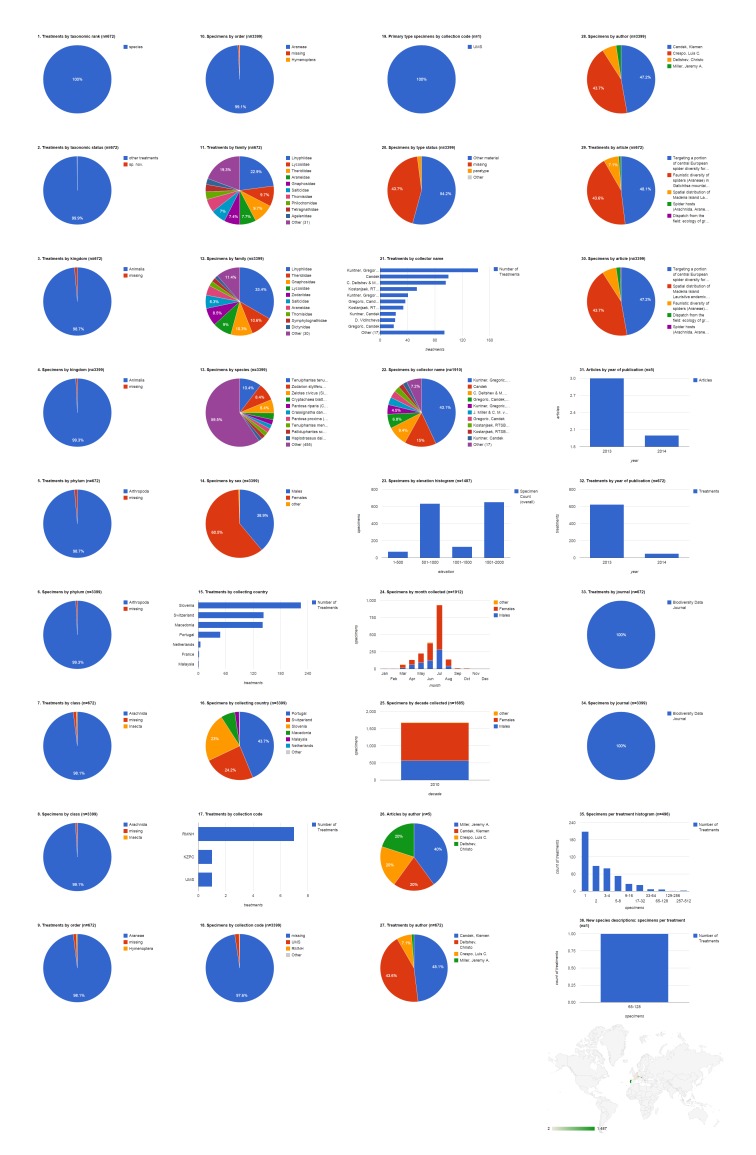
Species-rank treatments.

**Figure 5a.**
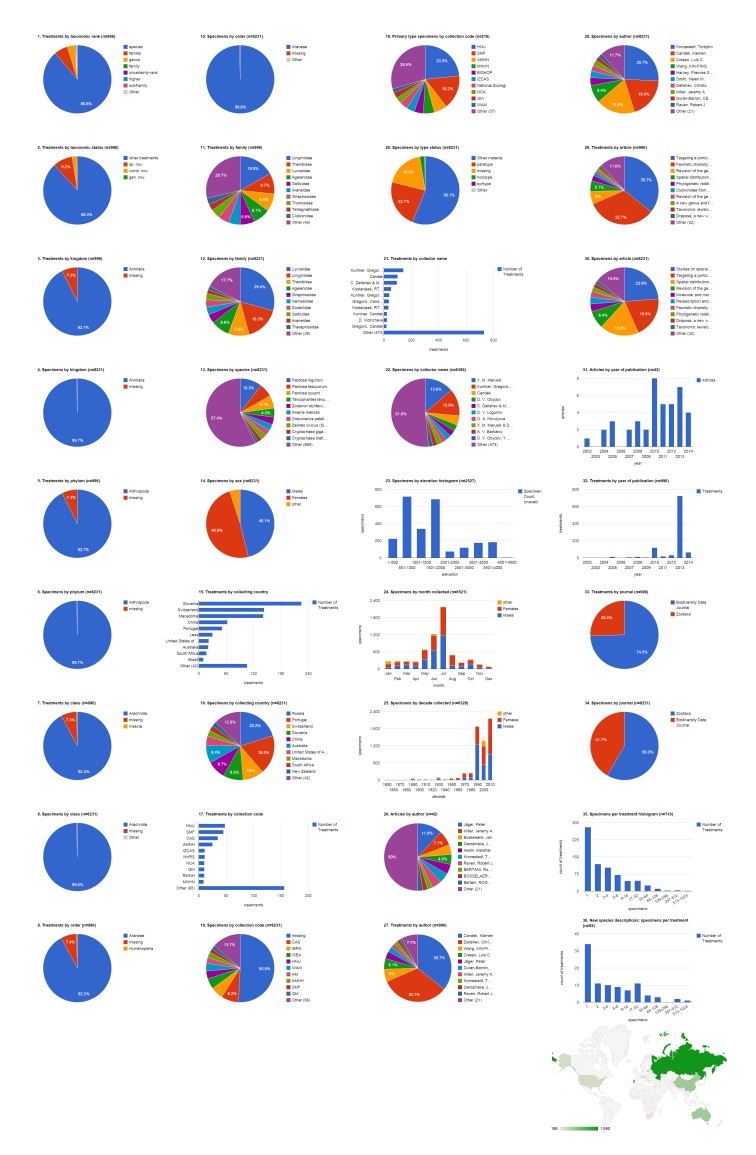
All treatments regardless of taxonomic rank.

**Figure 5b.**
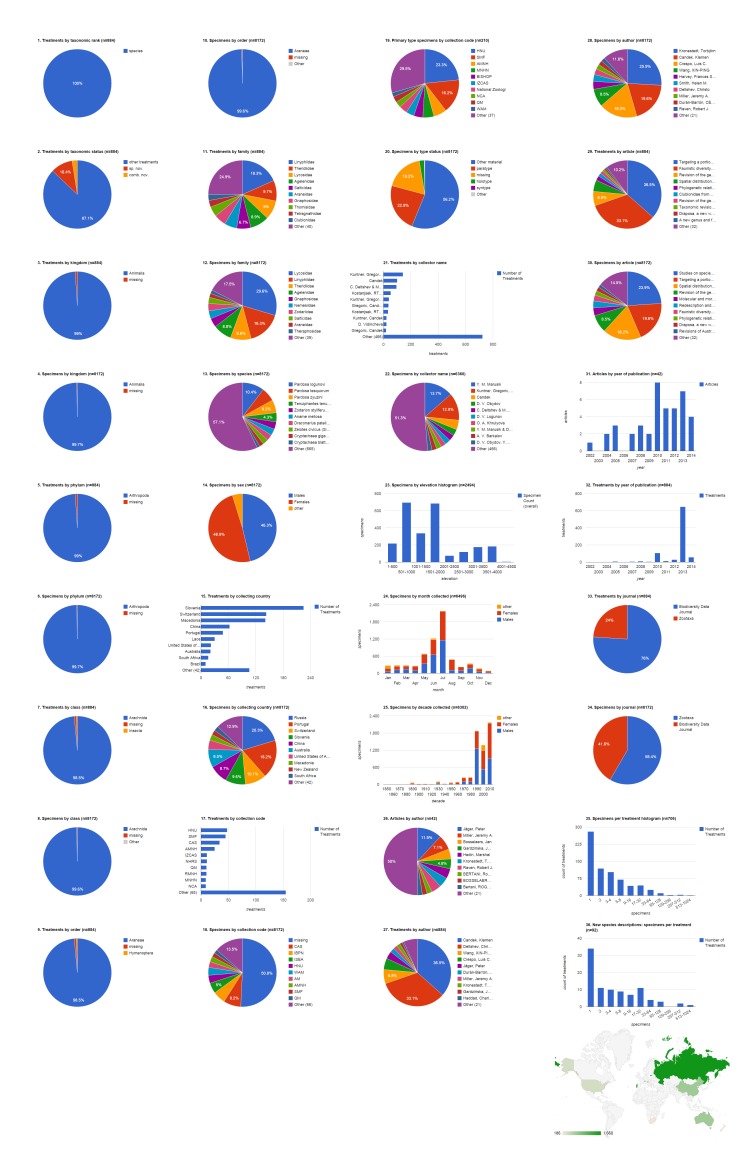
Species-rank treatments.

**Figure 9a.**
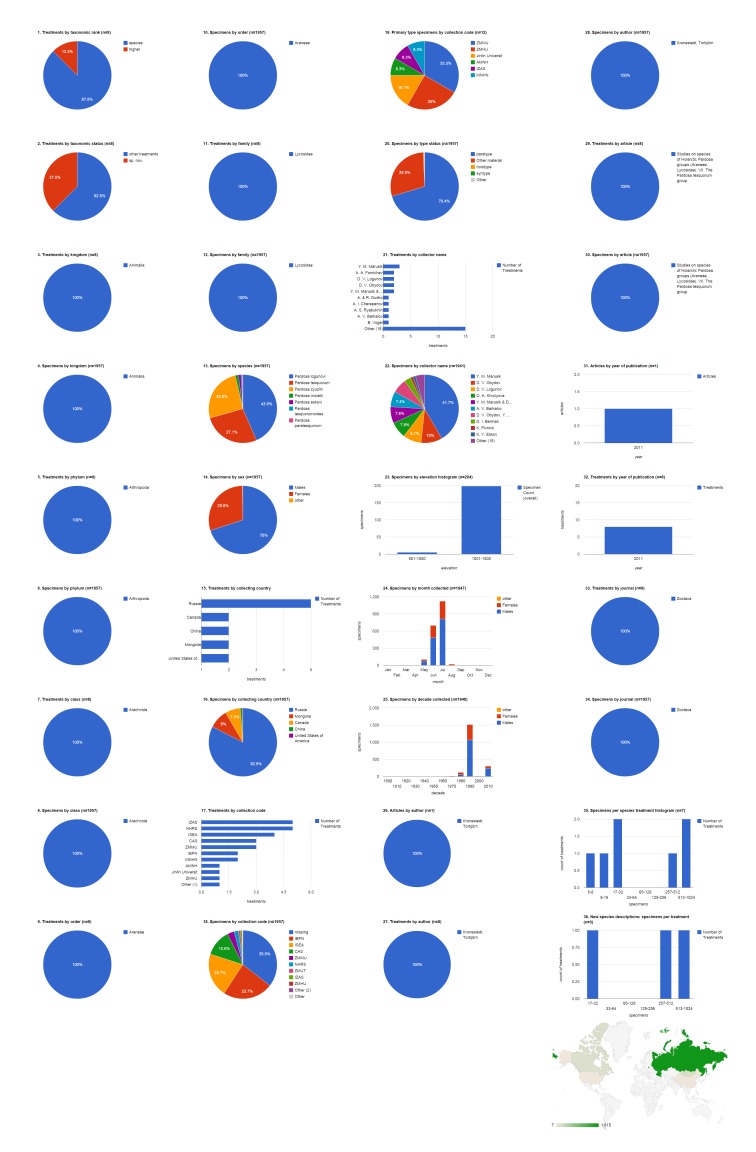
Data from all treatments in Kronestedt and Marusik (2011).

**Figure 9b.**
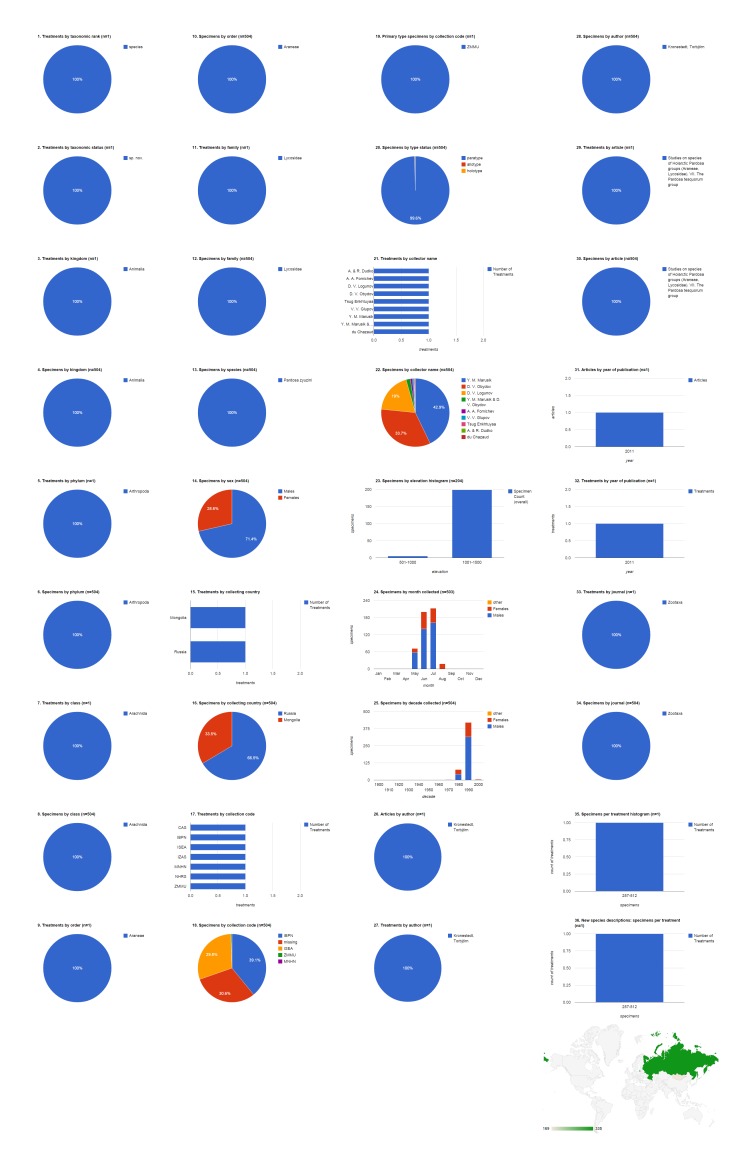
Data from one treatment, *Pardosa zyuzini* in Kronestedt and Marusik (2011).

**Table 1.** Open access articles in Zootaxa containing treatments on spiders (Araneae) as of August 2014. For each article, the page count, number of treatments (species rank, higher rank, and total), number of specimens, DOI, and Zoobank LSID (where available) are specified.

Source	Page count	Species treatments	Higher treatments	Total treatments	Specimens	DOI	Zoobank LSID
Cruz da Silva and Sierwald 2014	14	2	2	4	36	10.11646/zootaxa.3857.1.8	urn:lsid:zoobank.org:pub:8906CCE0-C5CC-4142-A9AF-98DA4BC953AF
Li et al. 2014	20	6	2	8	23	10.11646/zootaxa.3768.2.2	urn:lsid:zoobank.org:pub:B200DE30-D839-4107-A240-7DFB87A75635
Scharff and Hormiga 2013	4	1	1	2	1	10.11646/zootaxa.3750.2.8	urn:lsid:zoobank.org:pub:9515170F-60D0-43D3-A936-81E16EBEE6C3
Durán-Barrón et al. 2013	23	19	7	26	172	10.11646/zootaxa.3666.2.4	urn:lsid:zoobank.org:pub:FE211811-36E2-4A22-A55B-6E080E5CEC1D
Gardzińska and Patoleta 2013	6	4	1	5	13	10.11646/zootaxa.3664.1.4	urn:lsid:zoobank.org:pub:39F277A8-C7EB-4433-A87F-76A843CB1985
Patoleta and Gardzińska 2013	6	1	1	2	4	10.11646/zootaxa.3646.5.8	urn:lsid:zoobank.org:pub:50C87148-49F6-4BB2-BBF3-B5E6DD52B282
Smith et al. 2012	19	2	1	3	274	10.11646/zootaxa.3507.1.2	urn:lsid:zoobank.org:pub:8EDE33EB-3C43-4DFA-A1F4-5CC86DED76C8
Jäger 2012a	21	13	0	13	65	10.11646/33	
Harvey et al. 2012	24	4	1	5	304	10.11646/zootaxa.3383.1.3	
Raven 2012	25	6	2	8	118	10.11646/zootaxa.3305.1.2	
Jäger 2012b	8	3	0	3	3	10.11646/zootaxa.3228.1.3	
Kronestedt and Marusik 2011	34	7	1	8	1978	10.11646/zootaxa.3131.1.1	
Hedin and Carlson 2011	14	1	0	1	46	10.11646/zootaxa.2963.1.3	
Jäger and Praxaysombath 2011	4	1	0	1	4	10.11646/zootaxa.2883.1.5	
Bertani et al. 2011	18	2	1	3	15	10.11646/zootaxa.2814.1.1	
Vink et al. 2011	10	2	2	4	80	10.11646/zootaxa.2739.1.4	
Jäger and Dankittipakul 2010	21	13	3	16	41	10.11646/zootaxa.2730.1.2	
Kronestedt 2010	24	8	1	9	160	10.11646/zootaxa.2637.1.2	
Wang et al. 2010	127	60	0	60	730	10.11646/zootaxa.2593.1.1	
Jäger 2010	4	1	1	2	3	10.11646/zootaxa.2551.1.3	
Miller et al. 2010	36	9	2	11	133	10.11646/zootaxa.2534.1.1	
Gardzinska and Zabka 2010	17	7	4	11	6	10.11646/zootaxa.2526.1.2	
Bosselaers et al. 2010	11	1	0	1	2	10.11646/zootaxa.2427.1.3	
Haddad and Bosselaers 2010	12	7	1	8	54	10.11646/zootaxa.2361.1.1	
Lasut et al. 2009	4	1	0	1	45	10.11646/zootaxa.2267.1.5	
Bertani and Fukushima 2009	23	3	0	3	48	10.11646/zootaxa.2223.1.2	
Bertani et al. 2008	14	3	1	4	38	10.11646/zootaxa.1826.1.3	
Smith 2008	24	5	1	6	54	10.11646/zootaxa.1775.1.1	
Patrick et al. 2008	10	2	1	3	99	10.11646/zootaxa.1744.1.3	
Cokendolpher et al. 2007	12	3	1	4	39	10.11646/zootaxa.1529.1.4	
Opell et al. 2007	11	3	0	3	67	10.11646/zootaxa.1425.1.1	
Raven 2005	14	1	2	3	33	10.11646/zootaxa.1004.1.2	
Hedin and Dellinger 2005	19	5	0	5	80	10.11646/zootaxa.904.1.1	
Hendrixson and Bond 2005	19	2	2	4	51	10.11646/zootaxa.872.1.1	
Hendrixson and Bond 2004	14	1	0	1	42	10.11646/zootaxa.619.1.1	
Bosselaers 2004	8	1	0	1	1	10.11646/zootaxa.445.1.1	
Bosselaers and Henderickx 2002	8	1	0	1	10	10.11646/zootaxa.109.1.1	
