# Supplementary material for: Integrating and visualizing primary data from prospective and legacy taxonomic literature
Source: Biodivers Data J. 2015 May 12;(3):e5063. doi: 10.3897/BDJ.3.e5063 (PMC4442254; doi:10.3897/BDJ.3.e5063)
Supplement: Supplementary material 12 — Collector dashboard: specimens collected by Y. M. Marusik [file biodiversity_data_journal-3-e5063-s012.html]

Plazi dashboard: Collector & Taxonomic rank & Set of articles


**Plazi dashboard  
Collector = Y. M. Marusik &  
Taxonomic rank = species &  
Open access articles in Zootaxa containing treatments on spiders (Araneae) &  
Biodiversity Data Journal articles containing treatments on spiders (Araneae)**
