# Supplementary material for: Integrating and visualizing primary data from prospective and legacy taxonomic literature
Source: Biodivers Data J. 2015 May 12;(3):e5063. doi: 10.3897/BDJ.3.e5063 (PMC4442254; doi:10.3897/BDJ.3.e5063)
Supplement: Supplementary material 14 — Treatment dashboard: content from Pardosa zyuzini treatment in Kronestedt and Marusik (2011) [file biodiversity_data_journal-3-e5063-s014.html]

Plazi dashboard: Treatment


**Plazi dashboard  
Treatment = *Pardosa zyuzini* (http://treatment.plazi.org/id/BDA70EC9-F8AB-AED6-C2B7-628596A1714A) *in*  
Kronestedt & Marusik 2011 (DOI: 10.5281/zenodo.10109)**
