# Supplementary material for: Integrating and visualizing primary data from prospective and legacy taxonomic literature
Source: Biodivers Data J. 2015 May 12;(3):e5063. doi: 10.3897/BDJ.3.e5063 (PMC4442254; doi:10.3897/BDJ.3.e5063)
Supplement: Supplementary material 16 — Author dashboard: Jeremy A. Miller [file biodiversity_data_journal-3-e5063-s016.html]

Plazi dashboard: Author


**Plazi dashboard  
Articles in Biodiversity Data Journal and open access articles in Zootaxa containing treatments on spiders (Araneae) by Miller, Jeremy A. (lead author)**
