# Supplementary material for: Integrating and visualizing primary data from prospective and legacy taxonomic literature
Source: Biodivers Data J. 2015 May 12;(3):e5063. doi: 10.3897/BDJ.3.e5063 (PMC4442254; doi:10.3897/BDJ.3.e5063)
Supplement: Supplementary material 17 — Generating Interactive Dashboard Charts Based on Plazi Treatment Data [file biodiversity_data_journal-3-e5063-s017.docx]

**Generating Interactive Dashboard Charts Based on Plazi Treatment Data**

Charts are generated using javascripts that query Plazi’s Search and Retrieval (SRS) database and display the values received using the google charts API (<https://developers.google.com/chart/>). Source code for aggregation of XML structured treatment data on the Plazi server is available at <https://code.google.com/p/goldengate-server-docs/source/browse/#git%2Fsrc%2Fde%2Fuka%2Fipd%2Fidaho%2FgoldenGateServer%2Fdcs>​.

The first few lines of the chart include the chart’s unique identifier on the page (which must match the chart ID in domain 3, see below), establish style parameters, and point javascript to the google charts API. An example might look like this:

<div id=" chartDivxarTjRpOqoZbuIXu " style="width: 900px; height: 500px;">

<script type="text/javascript" src="https://www.google.com/jsapi"></script>

<script type="text/javascript"

This is followed by the main body of the javascript, which can be divided into three domains: 1) the chart type, series and value fields, 2) the filter, and 3) axes and labels. Domain 3 ends with chartID=”” with a unique text string between the quotation marks; this text string must match the one in quotes at the beginning of the div tag: <div id=””. The chart closes with:

></script>

</div>

We have created a repertoire of 37 standard charts for the dashboards. For each of the 37 charts, domains 1 and 3 remain constant, and the filter domain (2) changes with context and user input. There are a limited number of standard filter categories: Taxonomic group, Taxonomic rank, Taxonomic status, Journal, Country, Collection, Type status, Collector, Author, Article, and Treatment. Multiple filters can be applied simultaneously for compound queries involving multiple values of one category, and/or values of multiple categories. Additional filter categories are possible but are not implemented at this time. Some charts have an additional constant filter to remove placeholders for null values. Filters based on context and user input will be covered in section 2 “Filters”; section 1: “The Standard Charts” deals with the other aspects of each chart.

We currently have an unsolved problem with some axes and dealing with null values in an automated context. For example, some charts depict events in linear time (like number of specimens collected per decade). Ideally, the chart should display the number of specimens in each consecutive decade from the first collection to the most recent (including decades where the number of specimens collected is zero). But currently, for intermediate decades where the value is null, that decade is omitted from the chart unless we manually add that decade to an “artificial groups” field.

**The Standard Charts**

1. Treatments by taxonomic rank

*A pie chart with treatments proportioned by taxonomic rank*

Domain 1: src="http://plazi.cs.umb.edu/GgServer/srsStatCharts/chart.js?type=pie&series=tax.rank&seriesCutoff=10&field=DocCount&seriesOrder=-value

Domain 2:

Domain 3:

&emptySeriesLabelSubstitute=missing&title=1.%20Treatments%20by%20taxonomic%20rank&addDataSumToTitle=true&hAxis.logScale=false&hAxis.title=&vAxis.logScale=false&vAxis.title=&pieHole=0&region=world&artificialGroups=&isStacked=false&fontSize=16&chartId=

2. Treatments by taxonomic status

*A pie chart with treatments proportioned by taxonomic status*

Domain 1: src="http://plazi.cs.umb.edu/GgServer/srsStatCharts/chart.js?type=pie&series=tax.status&seriesCutoff=10&field=DocCount&seriesOrder=-value

Domain 2:

Domain 3:

&emptySeriesLabelSubstitute=other%20treatments&title=2.%20Treatments%20by%20taxonomic%20status&addDataSumToTitle=true&hAxis.logScale=false&hAxis.title=&vAxis.logScale=false&vAxis.title=&pieHole=0&region=world&artificialGroups=&isStacked=false&fontSize=16&chartId=

3. Treatments by kingdom

*A pie chart with treatments proportioned by taxonomic kingdom*

Domain 1: src="http://plazi.cs.umb.edu/GgServer/srsStatCharts/chart.js?type=pie&series=tax.kingdomEpithet&seriesCutoff=10&field=DocCount&seriesOrder=-value

Domain 2:

Domain 3:

&emptySeriesLabelSubstitute=missing&title=3.%20Treatments%20by%20kingdom&addDataSumToTitle=true&hAxis.logScale=false&hAxis.title=&vAxis.logScale=false&vAxis.title=&pieHole=0&region=world&artificialGroups=&isStacked=false&fontSize=16&chartId=

4. Specimens by kingdom

*A pie chart with specimens proportioned by taxonomic kingdom*

Domain 1: src="http://plazi.cs.umb.edu/GgServer/srsStatCharts/chart.js?type=pie&series=tax.kingdomEpithet&seriesCutoff=10&field=matCit.specimenCount&seriesOrder=-value

Domain 2:

Domain 3:

&emptySeriesLabelSubstitute=missing&title=4.%20Specimens%20by%20kingdom&addDataSumToTitle=true&hAxis.logScale=false&hAxis.title=&vAxis.logScale=false&vAxis.title=&pieHole=0&region=world&artificialGroups=&isStacked=false&fontSize=16&chartId=

5. Treatments by phylum

*A pie chart with treatments proportioned by taxonomic phylum*

Domain 1: src="http://plazi.cs.umb.edu/GgServer/srsStatCharts/chart.js?type=pie&series=tax.phylumEpithet&seriesCutoff=10&field=DocCount&seriesOrder=-value

Domain 2:

Domain 3:

&emptySeriesLabelSubstitute=missing&title=5.%20Treatments%20by%20phylum&addDataSumToTitle=true&hAxis.logScale=false&hAxis.title=&vAxis.logScale=false&vAxis.title=&pieHole=0&region=world&artificialGroups=&isStacked=false&fontSize=16&chartId=

6. Specimens by phylum

*A pie chart with specimens proportioned by taxonomic phylum*

Domain 1: src="http://plazi.cs.umb.edu/GgServer/srsStatCharts/chart.js?type=pie&series=tax.phylumEpithet&seriesCutoff=10&field=matCit.specimenCount&seriesOrder=-value

Domain 2:

Domain 3:

&emptySeriesLabelSubstitute=missing&title=6.%20Specimens%20by%20phylum&addDataSumToTitle=true&hAxis.logScale=false&hAxis.title=&vAxis.logScale=false&vAxis.title=&pieHole=0&region=world&artificialGroups=&isStacked=false&fontSize=16&chartId=

7. Treatments by class

*A pie chart with treatments proportioned by taxonomic class*

Domain 1: src="http://plazi.cs.umb.edu/GgServer/srsStatCharts/chart.js?type=pie&series=tax.classEpithet&seriesCutoff=10&field=DocCount&seriesOrder=-value

Domain 2:

Domain 3:

&emptySeriesLabelSubstitute=missing&title=7.%20Treatments%20by%20class&addDataSumToTitle=true&hAxis.logScale=false&hAxis.title=&vAxis.logScale=false&vAxis.title=&pieHole=0&region=world&artificialGroups=&isStacked=false&fontSize=

8. Specimens by class

*A pie chart with specimens proportioned by taxonomic class*

Domain 1: src="http://plazi.cs.umb.edu/GgServer/srsStatCharts/chart.js?type=pie&series=tax.classEpithet&seriesCutoff=10&field=matCit.specimenCount&seriesOrder=-value

Domain 2:

Domain 3:

&emptySeriesLabelSubstitute=missing&title=8.%20Specimens%20by%20class&addDataSumToTitle=true&hAxis.logScale=false&hAxis.title=&vAxis.logScale=false&vAxis.title=&pieHole=0&region=world&artificialGroups=&isStacked=false&fontSize=16&chartId=

9. Treatments by order

*A pie chart with treatments proportioned by taxonomic order*

Domain 1: src="http://plazi.cs.umb.edu/GgServer/srsStatCharts/chart.js?type=pie&series=tax.orderEpithet&seriesCutoff=10&field=DocCount&seriesOrder=-value

Domain 2:

Domain 3:

&emptySeriesLabelSubstitute=missing&title=9.%20Treatments%20by%20order&addDataSumToTitle=true&hAxis.logScale=false&hAxis.title=&vAxis.logScale=false&vAxis.title=&pieHole=0&region=world&artificialGroups=&isStacked=false&fontSize=16&chartId=

10. Specimens by order

*A pie chart with specimens proportioned by taxonomic order*

Domain 1: src="http://plazi.cs.umb.edu/GgServer/srsStatCharts/chart.js?type=pie&series=tax.orderEpithet&seriesCutoff=10&field=matCit.specimenCount&seriesOrder=-value

Domain 2:

Domain 3:

&emptySeriesLabelSubstitute=missing&title=10.%20Specimens%20by%20order&addDataSumToTitle=true&hAxis.logScale=false&hAxis.title=&vAxis.logScale=false&vAxis.title=&pieHole=0&region=world&artificialGroups=&isStacked=false&fontSize=16&chartId=

11. Treatments by family

*A pie chart with treatments proportioned by taxonomic family*

Domain 1: src="http://plazi.cs.umb.edu/GgServer/srsStatCharts/chart.js?type=pie&series=tax.familyEpithet&seriesCutoff=10&field=DocCount&seriesOrder=-value

Domain 2:

Domain 3:

&emptySeriesLabelSubstitute=missing&title=11.%20Treatments%20by%20family&addDataSumToTitle=true&hAxis.logScale=false&hAxis.title=&vAxis.logScale=false&vAxis.title=&pieHole=0&region=world&artificialGroups=&isStacked=false&fontSize=16&chartId=

12. Specimens by family

*A pie chart with specimens proportioned by taxonomic family*

Domain 1: src="http://plazi.cs.umb.edu/GgServer/srsStatCharts/chart.js?type=pie&series=tax.familyEpithet&seriesCutoff=10&field=matCit.specimenCount&seriesOrder=-value

Domain 2:

Domain 3:

&emptySeriesLabelSubstitute=missing&title=12.%20Specimens%20by%20family&addDataSumToTitle=true&hAxis.logScale=false&hAxis.title=&vAxis.logScale=false&vAxis.title=&pieHole=0&region=world&artificialGroups=&isStacked=false&fontSize=16&chartId=

13. Specimens by species

*A pie chart with specimens proportioned by taxonomic species*

Domain 1: src="http://plazi.cs.umb.edu/GgServer/srsStatCharts/chart.js?type=pie&series=tax.name&seriesCutoff=10&field=matCit.specimenCount&seriesOrder=-value

Domain 2:

Domain 3:

&emptySeriesLabelSubstitute=missing&title=13.%20Specimens%20by%20species&addDataSumToTitle=true&hAxis.logScale=false&hAxis.title=&vAxis.logScale=false&vAxis.title=&pieHole=0&region=world&artificialGroups=&isStacked=false&fontSize=16&chartId=

14. Specimens by sex

*A pie chart with specimens proportioned by sex*

Domain 1: src="http://plazi.cs.umb.edu/GgServer/srsStatCharts/chart.js?type=pie&field0=matCit.specimenCountMale&field0Aggregate=sum&field0Label=Males&field1=matCit.specimenCountFemale&field1Aggregate=sum&field1Label=Females&fieldSum=matCit.specimenCount&fieldSumLabel=other

Domain 2:

Domain 3:

&emptySeriesLabelSubstitute=&title=14.%20Specimens%20by%20sex&addDataSumToTitle=true&hAxis.logScale=false&hAxis.title=&vAxis.logScale=false&vAxis.title=&pieHole=0&region=world&artificialGroups=&isStacked=false&fontSize=16&buckets=&chartId=

15. Treatments by collecting country

*A bar chart with number of treatments containing records by country. Note: Data sum is not displayed with this chart*

Domain 1: src="http://plazi.cs.umb.edu/GgServer/srsStatCharts/chart.js?type=bar&field=DocCount&fieldAggregate=count-distinct&group=matCit.country&groupCutoff=10&groupOrder=-value&emptyGroupLabelSubstitute=

Domain 2:

&FILTER_matCit.country=a-

Domain 3:

&emptySeriesLabelSubstitute=&title=14.%20Specimens%20by%20sex&addDataSumToTitle=true&hAxis.logScale=false&hAxis.title=&vAxis.logScale=false&vAxis.title=&pieHole=0&region=world&artificialGroups=&isStacked=false&fontSize=16&buckets=&chartId=

16. Specimens by collecting country

*A pie chart with specimens proportioned by collecting country*

Domain 1: src="http://plazi.cs.umb.edu/GgServer/srsStatCharts/chart.js?type=pie&series=matCit.country&seriesCutoff=10&field=matCit.specimenCount&seriesOrder=-value

Domain 2:

Domain 3:

&emptySeriesLabelSubstitute=missing&title=16.%20Specimens%20by%20collecting%20country&addDataSumToTitle=true&hAxis.logScale=false&hAxis.title=&vAxis.logScale=false&vAxis.title=&pieHole=0&region=world&artificialGroups=&isStacked=false&fontSize=16&chartId=

17. Treatments by collection code

*A bar chart with number of treatments containing records by collection code. Note: Data sum is not displayed with this chart*

Domain 1: src="http://plazi.cs.umb.edu/GgServer/srsStatCharts/chart.js?type=bar&field=DocCount&fieldAggregate=count-distinct&group=matCit.collectionCode&groupCutoff=10&groupOrder=-value&emptyGroupLabelSubstitute=

Domain 2:

&FILTER_matCit.collectionCode=a-

Domain 3:

&emptySeriesLabelSubstitute=missing&title=17.%20Treatments%20by%20collection%20code&hAxis.logScale=false&hAxis.title=treatments&vAxis.logScale=false&vAxis.title=&pieHole=0&region=world&artificialGroups=&isStacked=false&fontSize=16&chartId=

18. Specimens by collection code

*A pie chart with specimens proportioned by collection code*

Domain 1: src="http://plazi.cs.umb.edu/GgServer/srsStatCharts/chart.js?type=pie&series=matCit.collectionCode&seriesCutoff=10&field=matCit.specimenCount&seriesOrder=-value

Domain 2:

Domain 3:

&emptySeriesLabelSubstitute=missing&title=18.%20Specimens%20by%20collection%20code&addDataSumToTitle=true&hAxis.logScale=false&hAxis.title=&vAxis.logScale=false&vAxis.title=&pieHole=0&region=world&artificialGroups=&isStacked=false&fontSize=16&chartId=

19. Primary type specimens by collection code

*A pie chart with primary type specimens proportioned by collection code*

Domain 1: src="http://plazi.cs.umb.edu/GgServer/srsStatCharts/chart.js?type=pie&series=matCit.collectionCode&seriesCutoff=10&field=matCit.specimenCount&seriesOrder=-value

Domain 2:

&FILTER_matCit.typeStatus=holotype syntype lectotype neotype

Domain 3:

&emptySeriesLabelSubstitute=missing&title=19.%20Primary%20type%20specimens%20by%20collection%20code&addDataSumToTitle=true&hAxis.logScale=false&hAxis.title=&vAxis.logScale=false&vAxis.title=&pieHole=0&region=world&artificialGroups=&isStacked=false&fontSize=16&chartId=

20. Specimens by type status

*A pie chart with specimens proportioned by type status*

Domain 1: src="http://plazi.cs.umb.edu/GgServer/srsStatCharts/chart.js?type=pie&series=matCit.typeStatus&seriesCutoff=10&field=matCit.specimenCount&seriesOrder=-value

Domain 2:

Domain 3:

&emptySeriesLabelSubstitute=missing&title=20.%20Specimens%20by%20type%20status&addDataSumToTitle=true&hAxis.logScale=false&hAxis.title=&vAxis.logScale=false&vAxis.title=&pieHole=0&region=world&artificialGroups=&isStacked=false&fontSize=16&chartId=

21. Treatments by collector name

*A bar chart showing the number of treatments collectors contributed material to. Note: Data sum is not displayed with this chart*

Domain 1: src="http://plazi.cs.umb.edu/GgServer/srsStatCharts/chart.js?type=bar&field=DocCount&fieldAggregate=count-distinct&group=matCit.collector&groupCutoff=10&groupOrder=-value&emptyGroupLabelSubstitute=

Domain 2:

&FILTER_matCit.collector=a-

Domain 3:

&title=21.%20Treatments%20by%20collector%20name&hAxis.logScale=false&hAxis.title=treatments&vAxis.logScale=false&vAxis.title=&pieHole=0&region=world&artificialGroups=&isStacked=false&fontSize=16&chartId=

22. Specimens by collector name

*A pie chart with specimens proportioned by collector name*

Domain 1: src="http://plazi.cs.umb.edu/GgServer/srsStatCharts/chart.js?type=pie&series=matCit.collector&seriesCutoff=10&field=matCit.specimenCount&seriesOrder=-value

Domain 2:

&FILTER_matCit.collector=a-

Domain 3:

&emptySeriesLabelSubstitute=missing&title=22.%20Specimens%20by%20collector%20name&addDataSumToTitle=true&hAxis.logScale=false&hAxis.title=&vAxis.logScale=false&vAxis.title=&pieHole=0&region=world&artificialGroups=&isStacked=false&fontSize=16&chartId=

23. Specimens by elevation histogram

*A column chart (histogram) showing the number specimens by elevation zone in 500 m increments*

Domain 1: src="http://plazi.cs.umb.edu/GgServer/srsStatCharts/chart.js?type=col&field=matCit.specimenCount&fieldAggregate=sum&group=matCit.elevation&groupCutoff=B&groupBuckets=0%3B500%3B1000%3B1500%3B2000%3B2500%3B3000%3B3500%3B4000%3B4500%3B5000%3B5500%3B6000%3B6500%3B7000%3B7500%3B8000%3B8500%3B9000&truncateGroupBuckets=true

Domain 2:

&FILTER_matCit.elevation=!-6366198

Domain 3:

&title=23.%20Specimens%20by%20elevation%20histogram&addDataSumToTitle=true&fontSize=16&isStacked=true&hAxis.logScale=false&hAxis.title=elevation&vAxis.logScale=false&vAxis.title=specimens&pieHole=0&region=world&chartId=

24. Specimens by month collected

*A column chart showing the number specimens by month collected*

Domain 1: src="http://plazi.cs.umb.edu/GgServer/srsStatCharts/chart.js?type=col&field0=matCit.specimenCountMale&field0Aggregate=sum&field0Label=Males&field1=matCit.specimenCountFemale&field1Aggregate=sum&field1Label=Females&fieldSum=matCit.specimenCount&fieldSumLabel=other&group=matCit.month&groupOrder=label

Domain 2:

&FILTER_matCit.month=!0

Domain 3:

&emptySeriesLabelSubstitute=&title=24.%20Specimens%20by%20month%20collected&addDataSumToTitle=true&hAxis.logScale=false&hAxis.title=month&vAxis.logScale=false&vAxis.title=specimens&pieHole=0&region=world&translateMonthNumbers=yes&artificialGroups=01%3B02%3B03%3B04%3B05%3B06%3B07%3B08%3B09%3B10%3B11%3B12&isStacked=true&fontSize=16&buckets=&chartId=

25. Specimens by decade collected

*A column chart showing the number specimens by decade collected. Note: artificialGroups used here to fill in null values for intermediate decades; modify as needed*

Domain 1: src="http://plazi.cs.umb.edu/GgServer/srsStatCharts/chart.js?type=col&field0=matCit.specimenCountMale&field0Aggregate=sum&field0Label=Males&field1=matCit.specimenCountFemale&field1Aggregate=sum&field1Label=Females&fieldSum=matCit.specimenCount&fieldSumLabel=other&group=matCit.decade&groupOrder=label

Domain 2:

&FILTER_matCit.decade=!0

Domain 3:

&emptySeriesLabelSubstitute=&title=25.%20Specimens%20by%20decade%20collected&addDataSumToTitle=true&hAxis.logScale=false&hAxis.title=decade&vAxis.logScale=false&vAxis.title=specimens&pieHole=0&region=world&artificialGroups=1860%3B1870&isStacked=true&fontSize=16&buckets=&chartId=

26. Articles by author

*A pie chart with articles proportioned by lead author*

Domain 1: src="http://plazi.cs.umb.edu/GgServer/srsStatCharts/chart.js?type=pie&series=bib.author&seriesCutoff=10&field=bib.title&seriesOrder=-value

Domain 2:

Domain 3:

&emptySeriesLabelSubstitute=&title=26.%20Articles%20by%20author&addDataSumToTitle=true&hAxis.logScale=false&hAxis.title=&vAxis.logScale=false&vAxis.title=&pieHole=0&region=world&artificialGroups=&isStacked=false&fontSize=16&buckets=&chartId=

<http://plazi.cs.umb.edu/GgServer/srsStatCharts/chart.js?type=pie&series=bib.author&seriesCutoff=10&field=DocCount&seriesOrder=-value>

27. Treatments by author

*A pie chart with treatments proportioned by lead author*

Domain 1:

src="http://plazi.cs.umb.edu/GgServer/srsStatCharts/chart.js?type=pie&series=bib.author&seriesCutoff=10&field=DocCount&seriesOrder=-value

Domain 2:

Domain 3:

&emptySeriesLabelSubstitute=&title=27.%20Treatments%20by%20author&addDataSumToTitle=true&hAxis.logScale=false&hAxis.title=&vAxis.logScale=false&vAxis.title=&pieHole=0&region=world&artificialGroups=&isStacked=false&fontSize=16&buckets=&chartId=

28. Specimens by author

*A pie chart with specimens proportioned by lead author*

Domain 1: src="http://plazi.cs.umb.edu/GgServer/srsStatCharts/chart.js?type=pie&series=bib.author&seriesCutoff=10&field=matCit.specimenCount&seriesOrder=-value

Domain 2:

Domain 3:

&emptySeriesLabelSubstitute=&title=28.%20Specimens%20by%20author&addDataSumToTitle=true&hAxis.logScale=false&hAxis.title=&vAxis.logScale=false&vAxis.title=&pieHole=0&region=world&artificialGroups=&isStacked=false&fontSize=16&chartId=

29. Treatments by article

*A pie chart with treatments proportioned by article*

Domain 1: src="http://plazi.cs.umb.edu/GgServer/srsStatCharts/chart.js?type=pie&series=bib.title&seriesCutoff=10&field=DocCount&seriesOrder=-value

Domain 2:

Domain 3:

&emptySeriesLabelSubstitute=missing&title=29.%20Treatments%20by%20article&addDataSumToTitle=true&hAxis.logScale=false&hAxis.title=&vAxis.logScale=false&vAxis.title=&pieHole=0&region=world&artificialGroups=&isStacked=false&fontSize=16&chartId=

30. Specimens by article

*A pie chart with specimens proportioned by article*

Domain 1: src="http://plazi.cs.umb.edu/GgServer/srsStatCharts/chart.js?type=pie&series=bib.title&seriesCutoff=10&field=matCit.specimenCount&seriesOrder=-value

Domain 2:

Domain 3:

&emptySeriesLabelSubstitute=missing&title=30.%20Specimens%20by%20article&addDataSumToTitle=true&hAxis.logScale=false&hAxis.title=&vAxis.logScale=false&vAxis.title=&pieHole=0&region=world&artificialGroups=&isStacked=false&fontSize=16&buckets=&chartId=

31. Articles by decade/year of publication

*A column chart showing the number articles by* decade/year *of publication. When the number of decades is small (>3), substitute year for series. Note: artificialGroups used here to fill in null values for intermediate decades/years; modify as needed*

*decade version:*

Domain 1: src="http://plazi.cs.umb.edu/GgServer/srsStatCharts/chart.js?type=col&field0=doc.articleUuid&field0Aggregate=count-distinct&field0Label=Articles&group=bib.decade&groupOrder=label

Domain 2:

Domain 3:

&emptySeriesLabelSubstitute=&title=31.%20Articles%20by%20decade%20of%20publication&addDataSumToTitle=true&hAxis.logScale=false&hAxis.title=decade&vAxis.logScale=false&vAxis.title=articles&pieHole=0&region=world&artificialGroups=2003%3B2006&isStacked=false&fontSize=16&buckets=&chartId=

*year version:*

Domain 1: src="http://plazi.cs.umb.edu/GgServer/srsStatCharts/chart.js?type=col&field0=doc.articleUuid&field0Aggregate=count-distinct&field0Label=Articles&group=bib.year&groupOrder=label

Domain 2:

Domain 3:

&emptySeriesLabelSubstitute=&title=31.%20Articles%20by%20year%20of%20publication&addDataSumToTitle=true&hAxis.logScale=false&hAxis.title=year&vAxis.logScale=false&vAxis.title=articles&pieHole=0&region=world&artificialGroups=&isStacked=false&fontSize=16&buckets=&chartId=

32. Treatments by year/decade of publication

*A column chart showing the number treatments by* decade/year *of publication. When the number of decades is small (>3), substitute year for series. Note: artificialGroups used here to fill in null values for intermediate decades/years; modify as needed*

*decade version:*

Domain 1: src="http://plazi.cs.umb.edu/GgServer/srsStatCharts/chart.js?type=col&field0=doc.uuid&field0Aggregate=count-distinct&field0Label=Treatments&group=bib.decade&groupOrder=label

Domain 2:

Domain 3:

&emptySeriesLabelSubstitute=&title=32.%20Treatments%20by%20decade%20of%20publication&addDataSumToTitle=true&hAxis.logScale=false&hAxis.title=year&vAxis.logScale=false&vAxis.title=treatments&pieHole=0&region=world&artificialGroups=&isStacked=false&fontSize=16&buckets=&chartId=

*year version:*

Domain 1: src="http://plazi.cs.umb.edu/GgServer/srsStatCharts/chart.js?type=col&field0=doc.uuid&field0Aggregate=count-distinct&field0Label=Treatments&group=bib.year&groupOrder=label

Domain 2:

Domain 3:

&emptySeriesLabelSubstitute=&title=32.%20Treatments%20by%20year%20of%20publication&addDataSumToTitle=true&hAxis.logScale=false&hAxis.title=year&vAxis.logScale=false&vAxis.title=treatments&pieHole=0&region=world&artificialGroups=2003%3B2006&isStacked=false&fontSize=16&buckets=&chartId=

33. Treatments by journal

*A pie chart with treatments proportioned by journal*

Domain 1: src="http://plazi.cs.umb.edu/GgServer/srsStatCharts/chart.js?type=pie&series=bib.source&seriesCutoff=10&field=DocCount&seriesOrder=-value

Domain 2:

Domain 3:

&emptySeriesLabelSubstitute=&title=33.%20Treatments%20by%20journal&addDataSumToTitle=true&hAxis.logScale=false&hAxis.title=&vAxis.logScale=false&vAxis.title=&pieHole=0&region=world&artificialGroups=&isStacked=false&fontSize=16&chartId=

34. Specimens by journal

*A pie chart with specimens proportioned by journal*

Domain 1: src="http://plazi.cs.umb.edu/GgServer/srsStatCharts/chart.js?type=pie&series=bib.source&seriesCutoff=10&field=matCit.specimenCount&seriesOrder=-value

Domain 2:

Domain 3:

&emptySeriesLabelSubstitute=&title=34.%20Specimens%20by%20journal&addDataSumToTitle=true&hAxis.logScale=false&hAxis.title=&vAxis.logScale=false&vAxis.title=&pieHole=0&region=world&artificialGroups=&isStacked=false&fontSize=16&chartId=

35. Specimens by treatment histogram

*A column chart (histogram) showing the number specimens per treatment on geometric scale*

Domain 1: src="http://plazi.cs.umb.edu/GgServer/srsStatCharts/chart.js?type=col&group=mat.specimenCount&groupCutoff=B&field=DocCount&fieldAggregate=sum&fieldOrder=label

Domain 2:

Domain 3:

&emptySeriesLabelSubstitute=&title=35.%20Specimens%20per%20treatment%20histogram&addDataSumToTitle=&hAxis.logScale=false&hAxis.title=specimens&vAxis.logScale=false&vAxis.title=count%20of%20treatments&pieHole=0&region=world&artificialGroups=&isStacked=true&fontSize=16&groupBuckets=1%3B2%3B4%3B8%3B16%3B32%3B64%3B128%3B256%3B512%3B1024%3B2048%3B4096%3B8192&truncateGroupBuckets=true&chartId=

36. New Species Descriptions Specimens per Treatment Histogram

*A column chart (histogram) showing the number specimens per new species treatment on geometric scale*

Domain 1: src="http://plazi.cs.umb.edu/GgServer/srsStatCharts/chart.js?type=col&group=mat.specimenCount&groupCutoff=B&field=DocCount&fieldAggregate=sum&fieldOrder=label

Domain 2:

Domain 3:

&emptySeriesLabelSubstitute=&title=36.%20New%20species%20descriptions:%20specimens%20per%20treatment&addDataSumToTitle=true&hAxis.logScale=false&hAxis.title=specimens&vAxis.logScale=false&vAxis.title=count%20of%20treatments&pieHole=0&region=world&artificialGroups=&isStacked=true&fontSize=16&groupBuckets=1%3B2%3B4%3B8%3B16%3B32%3B64%3B128%3B256%3B512%3B1024%3B2048%3B4096%3B8192&truncateGroupBuckets=true&chartId=

37. Map

*A map of specimens by country*

Domain 1: src="http://plazi.cs.umb.edu/GgServer/srsStatCharts/chart.js?type=geo&series=matCit.country&seriesCutoff=10&field=matCit.specimenCount&seriesOrder=-value

Domain 2:

Domain 3:

&emptySeriesLabelSubstitute=missing&title=36.%20Map&addDataSumToTitle=true&hAxis.logScale=false&hAxis.title=&vAxis.logScale=false&vAxis.title=&pieHole=0&region=world&artificialGroups=&isStacked=true&fontSize=16&chartId=

**Filters**

Contextual filters are part of Domain 2.

Filter: Taxonomic group

*Show only records for one or more selected species or higher taxa*

Taxonomic kingdom

&FILTER_tax.kingdomEpithet=

Example: &FILTER_tax.kingdomEpithet=Animalia

Taxonomic phylum

&FILTER_tax.phylumEpithet=

Example: &FILTER_tax.phylumEpithet=Arthropoda

Taxonomic class

&FILTER_tax.classEpithet=

Example: &FILTER_tax.classEpithet=Arachnida

Taxonomic order

&FILTER_tax.orderEpithet=

Example: &FILTER_tax.orderEpithet=Araneae

Taxonomic family

&FILTER_tax.familyEpithet=

Example: &FILTER_tax.familyEpithet=Lycosidae

Taxonomic species

&FILTER_tax.genusEpithet= &FILTER_tax.speciesEpithet=

&FILTER_tax.taxonomicName=

Example: &FILTER_tax.genusEpithet= Pardosa&FILTER_tax.speciesEpithet=logunovi

Example: &FILTER_tax.taxonomicName= Pardosa%20logunovi

Example: &FILTER_tax.taxonomicName= ‘’Pardosa logunovi’’

Filter: Taxonomic rank

*Show only records for one or more selected taxonomic ranks*

&FILTER_tax.rank=

Example: &FILTER_tax.rank=species

Filter: Taxonomic status

*Show only records for one or more selected taxonomic status values*

&FILTER_tax.status=

Example: &FILTER_tax.status=sp.%20nov.

Example: &FILTER_tax.status=’’sp. nov.’’

Filter: Journal

*Show only records for one or more selected journal*

&FILTER_bib.source=

Example: &FILTER_bib.source=%22Biodiversity%20Data%20Journal%22

Example: &FILTER_bib.source=’’Biodiversity Data Journal’’

Filter: Country

*Show only records for one or more selected countries*

&FILTER_matCit.country=

Example: &FILTER_matCit.country=Russia

&FILTER_matCit.country=Russia%20China

&FILTER_matCit.country=Russia China

&FILTER_matCit.country=%22United%20States%20of%20America%22

&FILTER_matCit.country=’’United States of America’’

Filter: Collection

*Show only records for one or more selected institutional collections*

&FILTER_matCit.collectionCode

Example: &FILTER_matCit.collectionCode=CAS

Filter: Type status

*Show only records for one or more selected type status*

&FILTER_matCit.typeStatus

Example: &FILTER_matCit.typeStatus=holotype

Filter: Collector

*Show only records for one or more selected collectors*

&FILTER_matCit.collector=

Example: &FILTER_matCit.collector=%22Y.%20M.%20Marusik%22

Example: &FILTER_matCit.collector=’’Y. M. Marusik’’

Filter: Author

*Show only records for one or more selected lead authors*

&FILTER_bib.author=

Example: & FILTER_bib.author=%22Miller,%20Jeremy%20A.%22

Example: & FILTER_bib.author=’’Miller, Jeremy A.’’

47. Filter: Article

*Show only records for one or more selected articles*

&FILTER_doc.name=

Example: &FILTER_doc.name=zt03131p034.xml

&FILTER_doc.name=zt00109.xml zt00445.xml zt00619.xml zt00872.xml zt00904.xml zt01004p028.xml zt01425p010.xml zt01529p060.xml zt01744p040.xml zt01775p024.xml zt01826p058.xml zt02223p047.xml zt02267p068.xml zt02361p012.xml zt02427p035.xml zt02526p053.xml zt02534p036.xml zt02551p068.xml zt02593p127.xml zt02637p054.xml zt02730p043.xml zt02739p050.xml zt02814p018.xml zt02883p068.xml zt02963p068.xml zt03131p034.xml zt03228p068.xml zt03305p052.xml zt03383p038.xml zt03415p057.xml zt03507p056.xml zt03646p592.xml zt03664p068.xml zt03666p193.xml zt03750p196.xml zt03768p138.xml zt03857p150.xml %22BiodivDatJour%201:e980%22 %22BiodivDatJour%201:e977%22 %22BiodivDatJour%201:e992%22 %22BiodivDatJour%202:%20e1051%22 %22BiodivDatJour%202:%20e1076%22

48. Filter: Treatment

*Show only records for one or more selected treatments*

&FILTER_doc.uuid=

Example: &FILTER_doc.uuid=BDA70EC9F8ABAED6C2B7628596A1714A
